# Four novel taxa of cyanobacteria from a unique thermal cave habitat in Vromoner Canyon, Albania

**DOI:** 10.1111/jpy.70082

**Published:** 2025-09-12

**Authors:** Jan Pokorný, Alžběta Vondrášková, Michaela Wipplingerová, Jan Kaštovský

**Affiliations:** ^1^ Department of Botany, Faculty of Science University of South Bohemia České Budějovice Czech Republic

**Keywords:** blue green algae, Cyanophyceae, evolution, phylogeny, taxonomy, warm spring

## Abstract

Thermal and cave habitats on nearly all continents have been a substantial source of new cyanobacterial genotypes and morphotypes that expanded with the dawn of the era of molecular phylogenetics. In this study, we investigated the cyanobacterial flora of an extreme habitat of recently discovered caves with sulfur‐rich thermal springs, using the polyphasic approach. The methods included cultivation, light and transmission electron microscopy, and molecular methods, including those that can be employed on samples that are not unialgal. Here, we present data on morphological and ultrastructural characteristics, 16S rRNA gene and 16S–23S internal transcribed spacer (ITS) rRNA region sequences, and folding structures. We identified one new trichal genus *Xomosiella* with the type species *X*. *audyi* forming a distinctly isolated clade and three new species in *Loriellopsis*, *Mastigocladus*, and *Pegethrix*. Apart from genetic distance, *Xomosiella* is distinguished from *Limnothrix* by its high trichome motility and benthic habitat, with granules likely composed of cyanophycin rather than aerotopes. The coccal cyanobacterium initially identified as “*Cyanosarcina*” sp. has been proposed as a new species, *Loriellopsis vromonerensis*, although its classification is complicated by morphological plasticity and phylogenetic uncertainties. The erection of *Mastigocladus boudae* was supported by a significant genetic divergence and distinct morphological characteristics. A description of a newly revealed cryptic species, *Pegethrix sulphurea*, has been provided. These results advance our knowledge of the diversity of cyanobacteria in extreme and understudied environments, which could enrich our understanding of microbial adaptability.

**Abbreviations**
AIC: Akaike information criterion
BA: Bayesian analysis
BBM: Bold Basal Medium
BI: Bayesian inference
CBFS: České Budějovice Faculty of Science Herbarium
CCALA: Culture Collection of Autotrophic Organisms at the Institute of Botany, Třeboň, CZ
DIC: differential interference contrast
DIV: D1′ index value
GTR: general time‐reversible
LM: light microscopy
ML: maximum likelihood
NCBI: National Center for Biotechnology Information
OTU: operational taxonomic unit
PCR: polymerase chain reaction
PD: phylogenetic diversity
SMS: Smart Model Selection
TEM: transmission electron microscopy

## INTRODUCTION

Cyanobacteria are a group of morphologically simple but diverse prokaryotic organisms with great ecological impact (Whitton, 2011). Modern molecular methods opened a new path from which to approach their taxonomy and evolution (Strunecký et al., 2022). Thus, discoveries of new taxa have risen over the last 2 decades and have reached dozens of descriptions annually (Kaštovský, 2023). One of the most frequent sources of scientifically novel taxa of cyanobacteria is extreme biotopes. In this work, we studied a locality with moderately thermal springs originating in caves with a high concentration of hydrogen sulfide.

In the past decade, numerous new taxa of thermophilic cyanobacteria with various morphologies and phylogenetic origins have been described from all over the world. Many new taxa with trichal morphotypes have originated from Asia (Cai et al., 2024; Jasser et al., 2022; Jiang et al., 2023; Tang et al., 2021, 2022), and Iranian radioactive thermal springs alone have given rise to four new genera and three new species of extant genera (Heidari et al., 2018). Yellowstone National Park showed previously uncharted diversity with 10 new species in one new and five extant genera (Kaštovský et al., 2023), and some new names have emerged from polyphasic revisions, for example, *Parathermosynechococcus lividus* or *Thermostichus* (Komárek et al., 2020; Tang et al., 2024). *Amphirytos*, *Kamptonema*, *Thermoleptolyngbya, Thermospirulina*, and a new species of *Haloleptolyngbya* have their origins in Europe (Bravakos et al., 2016; Cordeiro et al., 2024; Jasser et al., 2022; Moro et al., 2021; Sciuto & Moro, 2016; Strunecký et al., 2014). The heterocytous order Nostocales was expanded with the addition of the thermotolerant species of *Cyanocohniella* from Czechia (Kaštovský et al., 2014), *Mastigocladus ambikapurensis* from India (Jaiswal et al., 2021), and the new genus *Ewamiania* from Australia (McGregor & Sendall, 2017). A recently described thermophilic cyanobacterium from Africa is *Desertifilum fontinale* (Dadheech et al., 2014). New coccal species has been isolated and described de novo, for example, *Cryptococcum komarkovae* (Gama et al., 2019), *Gloeomargarita alhousahtiae* (Bacchetta et al., 2024), *Picosynechococccus fontinalis* (Komárek et al., 2020), or *Pseudochroococcus coutei* (Duval et al., 2021). Sometimes, the presence of new undescribed taxa has been proven by molecular methods without, however, being treated taxonomically (Bravakos et al., 2016; Momper et al., 2019; Tang et al., 2018).

Both natural and man‐made caves are sources of uncharted cyanobacterial diversity. Thirteen new species were identified from caves in Hawaii, including holotypes of the new genera *Goleter*, *Kovacikia*, *Pelatocladus*, and *Stenomitos* (Miscoe et al., 2016; Osorio‐Santos et al., 2014), and *Geitleria appalachiana* originated from continental North America (Kilgore et al., 2018). Several new taxa originated in Southern Europe. Most have come from Greece, that is, the genera *Iphinoë*, *Loriellopsis*, *Spelaeonaias*, *Speleotes*, *Speos*, and *Iphianassa* and the species *Stenomitos pantisii* and *Komarekiella chia* (Lamprinou et al., 2011, 2016; Panou & Gkelis, 2022). *Toxopsis calypsus* was described invalidly without a designated type (Lamprinou et al., 2012). *Albertania* and *Oculatella* were first isolated from catacombs in Malta and Rome, respectively (Zammit, 2018; Zammit et al., 2012). Two species of the hitherto unknown genus *Timaviella* were described from the Italian Giant Cave (Sciuto et al., 2017), and *Chalicogloea cavernicola* was described from Spain (Roldán et al., 2013). *Cylindrospermum moravicum* was isolated from cave sediment in Czechia (Moravian Karst; Johansen et al., 2014) and a cave species of *Roholtiella*, *R*. *mixta,* from Russia (Abdullin et al., 2021).

From habitats rich in hydrogen sulfide (H_2_S), very few taxa have been described in the 21st century, and only thanks to new combinations, for example, *Phormidium caerulescens* (Anagnostidis, 2001; Gicklhorn, 1921; Komárek & Anagnostidis, 2008) and several species of *Kamptonema* (Strunecký et al., 2014). Cyanobacteria in such habitats may be influenced by the detrimental effect of H_2_S on photosynthesis (Lumian et al., 2021). However, some cyanobacteria can cope with these conditions; thus, their diversity is important to characterize (Miller & Bebout, 2004).

## MATERIALS AND METHODS

### Locality

The Vromoner Canyon is located on the Albanian–Greek border, 9 km southeast of the town of Leskovik, southeastern Albania. It was formed by the Sarandoporo River, which then flows into the Vjosa River after 8 km. The dominant rock is limestone, and the area falls within the Kruja thermal water province (Eftimi & Frashëri, 2018). There are several small thermal springs in the area, which have been used as spas since ancient times. The water temperature is between 26 and 28°C, and the hydrogen sulfide content is high, 40–65 mg · L^−1^. The waters have higher contents of sodium (Na), chlorine (Cl), lithium (Li), and fluorine (F), and relatively low concentrations of calcium (Ca) and hydrogen carbonate (HCO_3_; Audy et al., 2022). The concentration of free hydrogen sulfide in the air often reaches toxic levels and poses a problem for local research. These springs have formed a series of semi‐thermal cave systems, which are very specific both geologically and biologically. They were first discovered in 2022 (Audy et al., 2022; Sarbu et al., 2024), and the cyanobacteria for the present paper were collected during a speleological expedition organized by the Czech Speleological Society, ZO 6–17 Topas from October 27 to November 9, 2022.

### Morphological studies and cultivation

The collected samples were immediately investigated in the field using an Olympus CX21 LED microscope (Olympus Corp., Tokyo, Japan) with a digital camera (InfinityX‐32, Lumenera Corporation, Canada) and the software Infinity Analyze (Lumenera Corporation, Canada). The most viable samples were transferred into 12‐well Nunc™ Cell‐Culture Treated Multidishes (ThermoFisher Scientific, Massachusetts, United States) with Z8 medium (Staub, 1961), Bold Basal Medium (BBM, Bischoff & Bold, 1963; Bold, 1949), and D medium (Sheridan, 1966) solidified with 1.5% agarose and culture tubes with liquid BBM and transported to the phycological laboratory of the University of South Bohemia in České Budějovice (with permission of Ministry of Tourism and Environment of Republic of Albania No. 416, according to Nagoya protocol). The crude cultures were later grown on corresponding solid and liquid media. The unialgal strains were isolated and purified using a combination of streaking on agar plates and isolation using a cultivation needle and a glass microcapillary. The unialgal strains were grown in cultivation tubes with liquid medium, while the morphology was documented using the Olympus BX 51 light microscope (400× and 1000× magnification) with Nomarski DIC (Olympus Corp., Tokyo, Japan), the DP–74 digital camera, and the Olympus cellSens Entry software (Olympus Corp., Tokyo, Japan). For the molecular methods, the cultured biomass was isolated using a glass microcapillary, washed, and stored in a droplet of TE buffer in a 0.2‐mL polymerase chain reaction (PCR) tube (Jung et al., 2024; Mareš et al., 2015; Zapomělová et al., 2007). Part of the biomass of the strains Alb61‐JP1 and Alb16‐JP1 was also harvested in individual 2‐mL PCR tubes for observation by transmission electron microscopy.

### Electron microscopy

To note ultrastructural features mainly focusing on thylakoid position, specimens were preserved in 2.5% glutaraldehyde and 0.1 M cacodylate buffer followed by post‐fixation with 2% osmium tetroxide. The fixed material was dehydrated in an acetone series (30%, 50%, 70%, 80%, 90%, 95%, and 100%) and embedded in Spurr's resin (Spurr, 1969). The 70‐nm ultrathin sections were placed onformvar‐coated grids, contrasted by uranyl acetate and lead citrate, and analyzed by a JEOL JEM 1010 microscope in the Laboratory of Electron Microscopy, Biology Centre ASCR—Institute of Parasitology, České Budějovice, Czech Republic.

### Molecular analysis

For the strains Alb16‐JP1, Alb61‐JP1, and Alb62‐JP1, direct PCR was carried out (Jung et al., 2024). The biomass isolated for molecular methods was first lysed using the Platinum Direct PCR Universal Master Mix kit (Thermo Fisher Scientific, Waltham, MA, United States) following a modified manufacturer protocol (Jung et al., 2024). Targeting the 16S rRNA gene and adjacent 16S–23S ITS rRNA region, PCR was performed in the Biometra T3000 thermocycler (Analytik Jena GmbH, Jena, Germany). For the PCR, 2 μL of the lysate was used as a template. The reaction consisted of 6 μL dH_2_O, 10 μL Platinum Direct PCR Universal Master Mix, 5 pmol of primer VRF2F (5′– GGG GAA TTT TCC GCAATG GG –3′; Johansen et al., 2011 after Nübel et al., 1997), and 5 pmol of primer VRF1R (5′– CTC TGT GTG CCT AGG TAT CC –3′; Johansen et al., 2011 after Wilmotte et al., 1993). The amplification started with denaturation at 94°C for 2 min, continued with 40 cycles of denaturation at 94°C for 15 s, primer annealing at 60°C for 15 s, and elongation at 68°C for 5 min and 30 s, ending with the final elongation at 68°C for 10 min. The success of PCR product amplification was evaluated by 1.5% agarose gel electrophoresis and visualization in the GenoSens 2000 transilluminator (Clinx Science Instruments Co., Ltd, Shanghai, China), purified using ExoSAP‐IT® (Bell, 2008), and sequenced commercially using Sanger sequencing (SEQme s.r.o., Dobříš, Czech Republic). Both PCR primers, as well as the inner primer—VRF7F (5′– AAT GGG ATT AGA TAC CCC AGT AGT C –3′; Patzelt et al., 2014 after Nübel et al., 1997), VRF5F (5′– TGT ACA CAC CGG CCC GTC –3′; Johansen et al., 2011 after Wilmotte et al., 1993) or VRF6R (5′– GAC GGG CGG TGT GTA CA –3′; Johansen et al., 2011 after Wilmotte et al., 1993)—were used if necessary to ensure a complete purity of the sequence.

Because the strain Alb13‐JP1 grew very slowly, and to compare the morphology of the natural coccal population from the crude culture Alb16 with the unialgal strain, we used a cultivation‐independent approach based on single colonies. Thus, the targeted 16S rRNA gene and 16S–23S ITS rRNA regions were amplified using multiple strand displacement amplification (Lara et al., 2013; Rodrigue et al., 2009; Spits et al., 2006) and the following PCR, as well as the direct two‐step PCR approach according to the protocol of Pokorný et al. (2023) following previous publications (Lara et al., 2013; Mareš et al., 2015). The colonies and filaments respectively were isolated using glass microcapillaries and washed in several drops of TE‐buffer and were observed and checked for possible contamination using an Olympus CX40 microscope at 200× magnification before they were placed in the 0.2‐mL PCR tubes. Successfully amplified PCR products were also purified using ExoSAP‐IT® and sequenced using Sanger sequencing.

To capture the possible variability of the ribosomal operons, the PCR products of Alb13‐JP1, Alb13‐JP2, and Alb61‐JP1 were also cloned into *Escherichia coli* DH5‐Alpha cells using the pGEM®‐T Easy vector system (Promega Corp., Madison, WI, United States) following the standard procedure (Johansen et al., 2017). Colonies with ligated PCR products were denatured in 40 μL of dH_2_O and thereafter used as a template for PCR consisting of 6.8 μL dH_2_O, 10 μL 2× Plain PP Master Mix, 4 pmol primer T7 (5′– TAA TAC GAC TCA CTA TAG GG −3′), 4 pmol primer SP6 (5′– ATT TAG GTG ACA CTA TAG −3′), and 2 μL denaturation product as a template in a Biometra T3000 thermocycler starting with denaturation at 95°C for 5 min followed by 45 cycles of denaturation at 94°C for 1 min, primer annealing at 52°C for 45 s, and elongation at 72°C for 2 min ending with the final elongation at 72°C for 10 min. The PCR products were treated and directly sequenced as described above, using sp6 and T7 primers.

The sequences were assembled using Geneious Prime 2024.0.5 (https://www.geneious.com) and error proofed by eye. The assembled sequences were aligned with additional relevant sequences recovered using BLAST® (Altschul et al., 1990), the National Center for Biotechnology Information (NCBI) GeneBank database (Sayers et al., 2022), and CyanoSeq 1.1.1 (Lefler et al., 2023), following the cyanobacterial system revised by Strunecký et al. (2022) and the recent literature. The final alignments were constructed using Geneious Prime 2024.0.5 with the MAFFT algorithm (Katoh et al., 2002; Katoh & Standley, 2013) and adjusted manually.

Before the final phylogenetic trees were calculated, a large dataset was used to infer the mutual relationship of the investigated strains (data not shown). These results were then used to choose the sequences accompanying the sequences of our strains in the final datasets. Maximum likelihood (ML) analysis with 1000 bootstrap replicates was carried out using the PhyML online server (ngphylogeny.fr), with the best model chosen by Smart Model Selection (SMS) using the Akaike information criterion (AIC; Guindon et al., 2010; Lefort et al., 2017; Lemoine et al., 2018, 2019). Bayesian inference (BI) was performed using MrBayes 3.2.6 (Huelsenbeck & Ronquist, 2001) implemented in Geneious Prime 2024.0.5, and the analysis spanned 10 million generations sampling every 1000 generations until the average standard deviation of split frequencies was below 0.01. The presented phylogenetic tree topologies were inferred from ML analyses using FigTree 1.4.4 (http://tree.bio.ed.ac.uk/software/figtree/) and graphically processed in Inkscape 1.2.1 (https://inkscape.org).

The semi‐conserved structures of the 16S–23S ITS rRNA region motifs were identified by comparative analysis with the closest related taxa following Boyer et al. (2001) and Iteman et al. (2000). When present, the D1‐D1′, V2, Box‐B, and V3 structures were visualized using mFold RNA (unafold.org) folding form (Markham & Zuker, 2008; Zuker, 2003) and compared by eye. In all cases, the ITS rRNA region sequences were too divergent from the closest related taxa; therefore, we were unable to align them and calculate base pair substitution rates with confidence. The 16S rRNA p‐distance matrices were formed using MEGA11 with default settings and recalculated for phylogenetic diversity (PD) values (p‐distance × 100). To further highlight the divergence among the pseudo‐cryptic species of *Pegethrix*, the D1′ index was calculated, and the values (DIV) were plotted against the total length of the D1‐D1′ helix (Villanueva et al., 2024).

## RESULTS

Voucher specimens have been deposited in an institution recognized by Index Herbariorum (Thiers, 2025), the Herbarium of the University of South Bohemia, České Budějovice, Czech Republic (CBFS).

### Taxonomic descriptions

#### *Xomosiella* J.Pokorný & Kaštovský gen. nov.

**Etymology:** Named after ancient spa Xomos, where the cyanobacterium was isolated.

**FIGURE 1:**
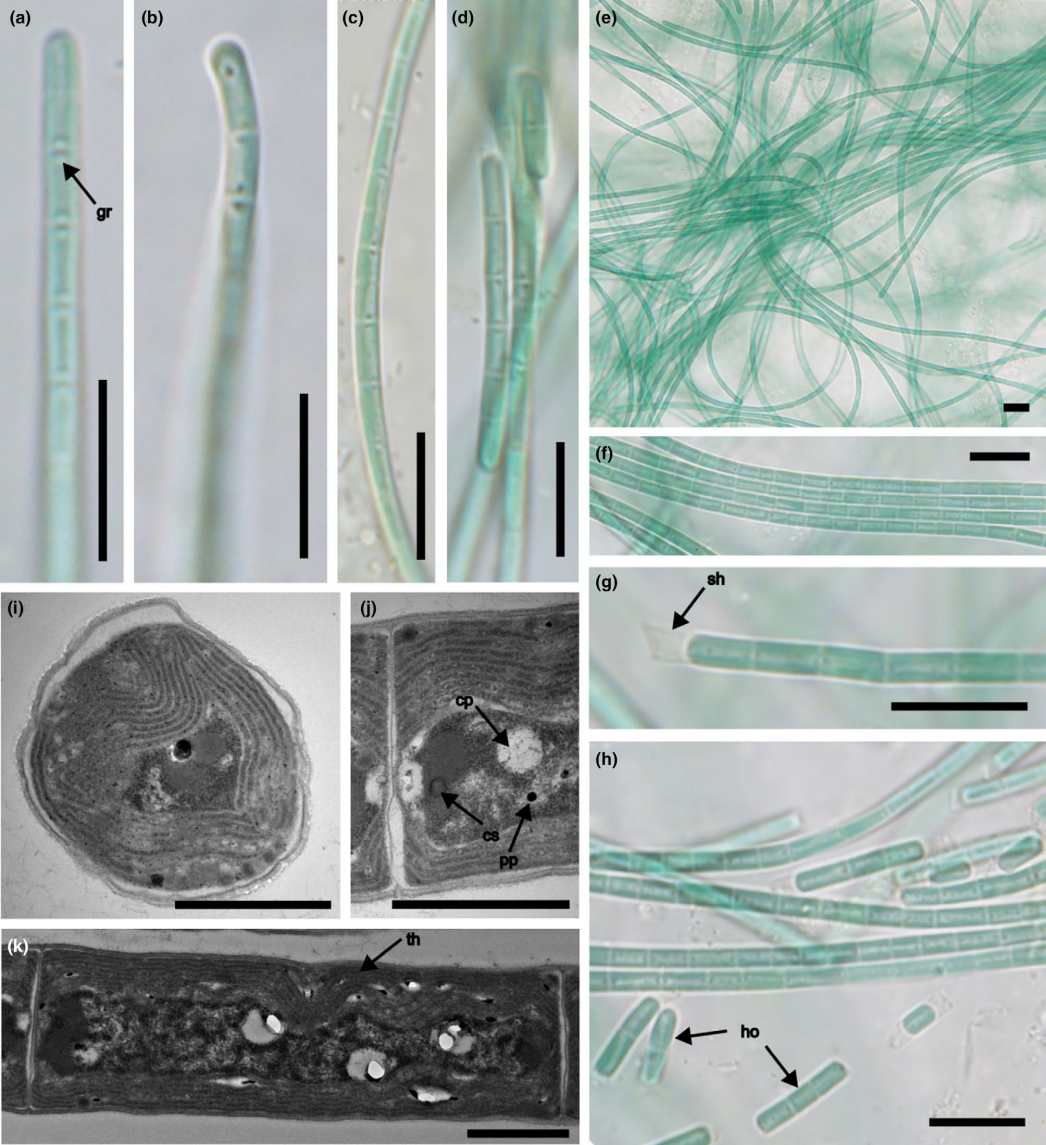
The overall morphology of *Xomosiella audyi*, strains Alb61‐JP1 (a–d, i–k) and Alb62‐JP1 (e–h). Light microscopy micrographs: apexes of individual mature filaments (a, b), middle part of the filament (c) with hormogonia (d), bundles of filaments (e, f) with hormogonia (h), broken filament with visible sheath (g); transmission electron microscopy micrographs: transversal (i) and longitudinal (j, k) section of the filament with visible thylakoid arrangement and polar granules detail. gr—granules, sh—sheath, cp—cyanophycin, cs—carboxysome, pp—polyphosphate, th—thylakoids, ho—hormogonia. Scale bars 10 μm (a–h); 1 μm (i–k).

**Type species:** *Xomosiella audyi* J.Pokorný & Kaštovský sp. nov. (Figure 1)

**Diagnosis:** The genus differs by high motility and very fine sheaths sometimes visible after trichome breakup, which are absent in *Limnothrix* sensu stricto. Phylogenetically distinct and placed in Persinemataceae, *Xomosiella* clearly differs molecularly by the distinct 16S rRNA gene and 16S–23S ITS rRNA region sequence (GenBank accession number PQ651705 of strain Alb61‐JP1 clone 2) and the secondary structure of its conserved motifs.

**Description:** Colony pale or dark blue‐green, filaments pale blue‐green, simple, uniseriate, straight or slightly wavy, isopolar, without branching, and intensively motile. Trichomes many‐celled, straight or bent. Thin sheaths colorless, apparent only in some filaments (Figure 1g). Trichomes less than 3 μm wide, not constricted at cross‐walls, which are clearly visible. Cells cylindrical with parietal thylakoids, unconstricted, longer than wide (after cell divison more or less isodiametric), 1.8–2.5 μm wide (2.15 ± 0.4) and 3–6 μm long (4.55 ± 1.45), often with one or two prominent propagules near cross‐walls. Terminal cells rounded, not tapering, without calyptra. Division without necridic cells.

**Holotype:** CBFS A‐260, dried material of the reference strain CCALA 10344 preserved on paper in a metabolically inactive state, kept at CBFS, *leg*. M. Wipplingerová and J. Kaštovský, 2 Nov. 2022, originally growing in a benthic mat of a warm pool

**Type locality:** Ruins of old spa Xomos, Vromoner Canyon, Albania (40°5′54.073″ N, 20°40′23.998″ E)

**Ecology:** Benthic, mat‐forming in semi‐thermal sulfuric water, 28°C, less abundant component of massive growth of *Spirulina* sp.

**Etymology:** *audyi*—named to honor Marek Audy, speleologist, who co‐organized this expedition

**Reference strain:** CCALA 10344 (housed at Culture Collection of Autotrophic Organisms at the Institute of Botany, Třeboň, CZ), isolated as Alb61‐JP1 by J. Pokorný

#### *Loriellopsis vromonerensis* Kaštovský, J.Pokorný & Vondrášková sp. nov. (Figure 2)

**FIGURE 2:**
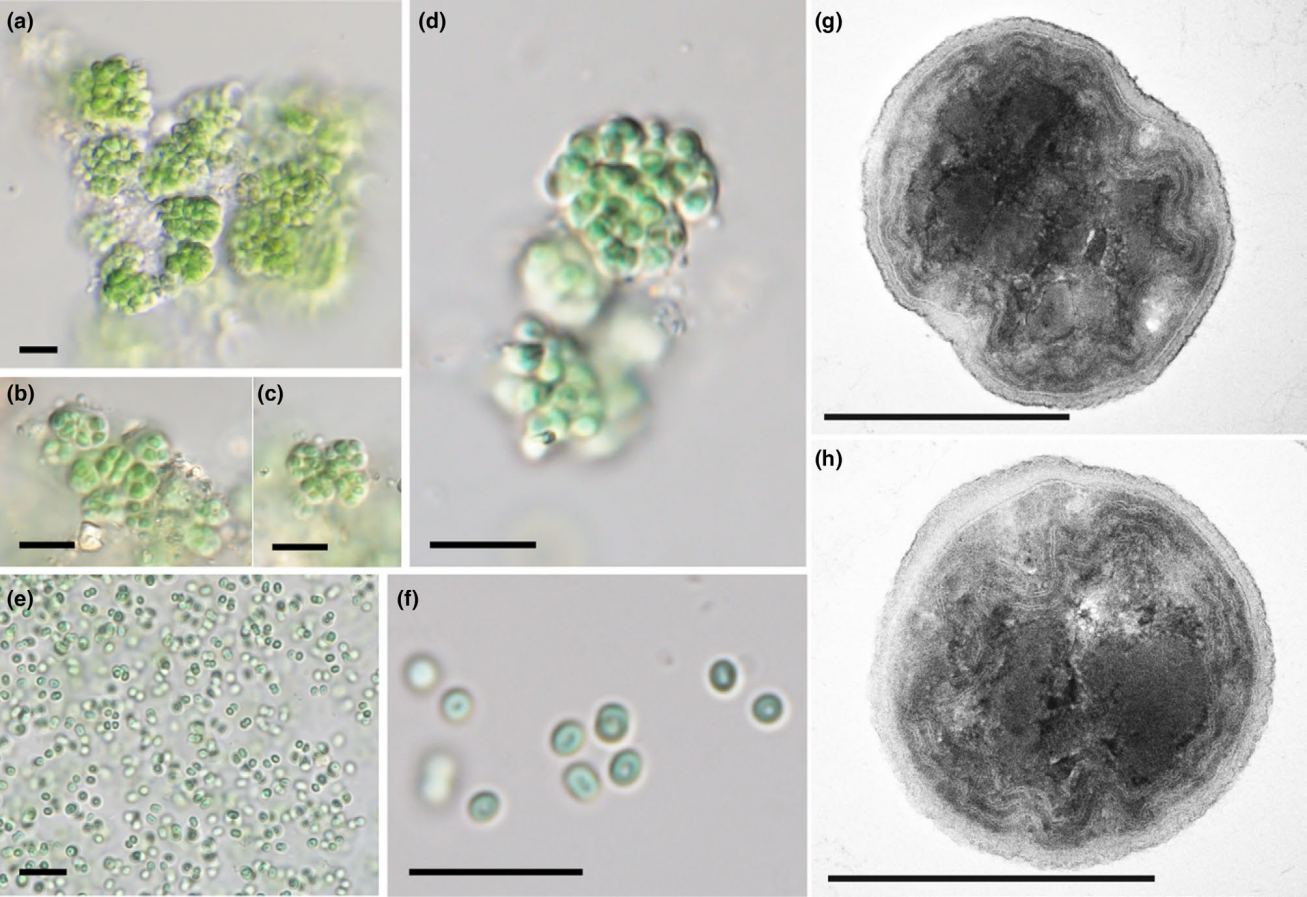
The overall morphology of *Loriellopsis vromonerensis*. Light microscopy micrographs: colonies from the crude culture of sample Alb16 (a–d), loose aggregates (e) and cell tetrads (f) from the strain Alb16‐JP1; transmission electron microscopy micrographs: transversal section of the cells of the strain Alb16‐JP1 (g, h). Scale bars 10 μm (a–f), 1 μm (g, h).

**Diagnosis:** Within the genus *Loriellopsis*, it is clearly distinguished by the fact that it never forms any filaments, not even the true‐branched ones that are typical for this genus. The morphology of the thalus is coccal, forming sarcinoid packages. It is morphologically cryptic to the genus *Cyanosarcina*. *Loriellopsis vromonerensis* is characterized by its unique sequences for the 16S rRNA gene and 16S–23S ITS rRNA region (GenBank accession number PQ773165 of strain Alb16‐JP1).

**Description:** Coccal organism, cells gathered in irregular microscopic, later macroscopic colonies with sarcinoid organization. Colonies dark blue green, lumpy, composed of microscopic bundles of cells enveloped in colorless common mucilage; bundles 4–22 μm broad (13 ± 9), often aggregate in larger bulks; clear tetrads are sometimes visible with their own mucilaginous envelope, in culture, forming macroscopic aggregates. Each bundle contains eight (>100) cells. Individual cells bright blue green to olive green, oval to irregularly polyhedral, rarely spherical 1.3–3.7 μm wide (2.5 ± 1.2), with thin individual gelatinous envelopes. Cell content homogeneous with darker edges, rarely facultatively granulated. Thylakoids parietal with occasional protrusions toward the center of the cell. Common mucilage up to 2 μm thick, lamellated. No resting stages or other structures were observed. Under culture conditions, the aggregates disintegrate, losing common mucilage, cells are loosely dispersed, spherical, oval, or subspherical, occasionally forming loose tetrads.

**Holotype:** CBFS A‐261, dried material of a crude culture with dominant presence of herein described taxon accompanied by unidentified coccal green alga, preserved on paper in a metabolically inactive state, kept at CBFS, *leg*. M. Wipplingerová and J. Kaštovský, 1 Nov. 2022, originally growing atmophytically on a cave wall in the entrance zone

**Type locality:** Entrance zone of Sulfur Cave, Vromoner Canyon, Greece/Albania border (40°5′46.539″ N, 20°40′43.841″ E)

**Ecology:** Macroscopic colony on the cave ceiling above the underground lake, water with high content of hydrogen sulfide, with low illuminance. A relatively abundant species at the locality

**Etymology:** *Vromonerensis—*named based on the name of the locality of Vromoner canyon

**Reference strain:** CCALA 10343 (housed at Culture Collection of Autotrophic Organisms at the Institute of Botany, Třeboň, CZ), isolated as Alb16‐JP1 by J. Pokorný

#### *Mastigocladus boudae* Kaštovský, J.Pokorný & Vondrášková sp. nov. (Figure 3)

**FIGURE 3:**
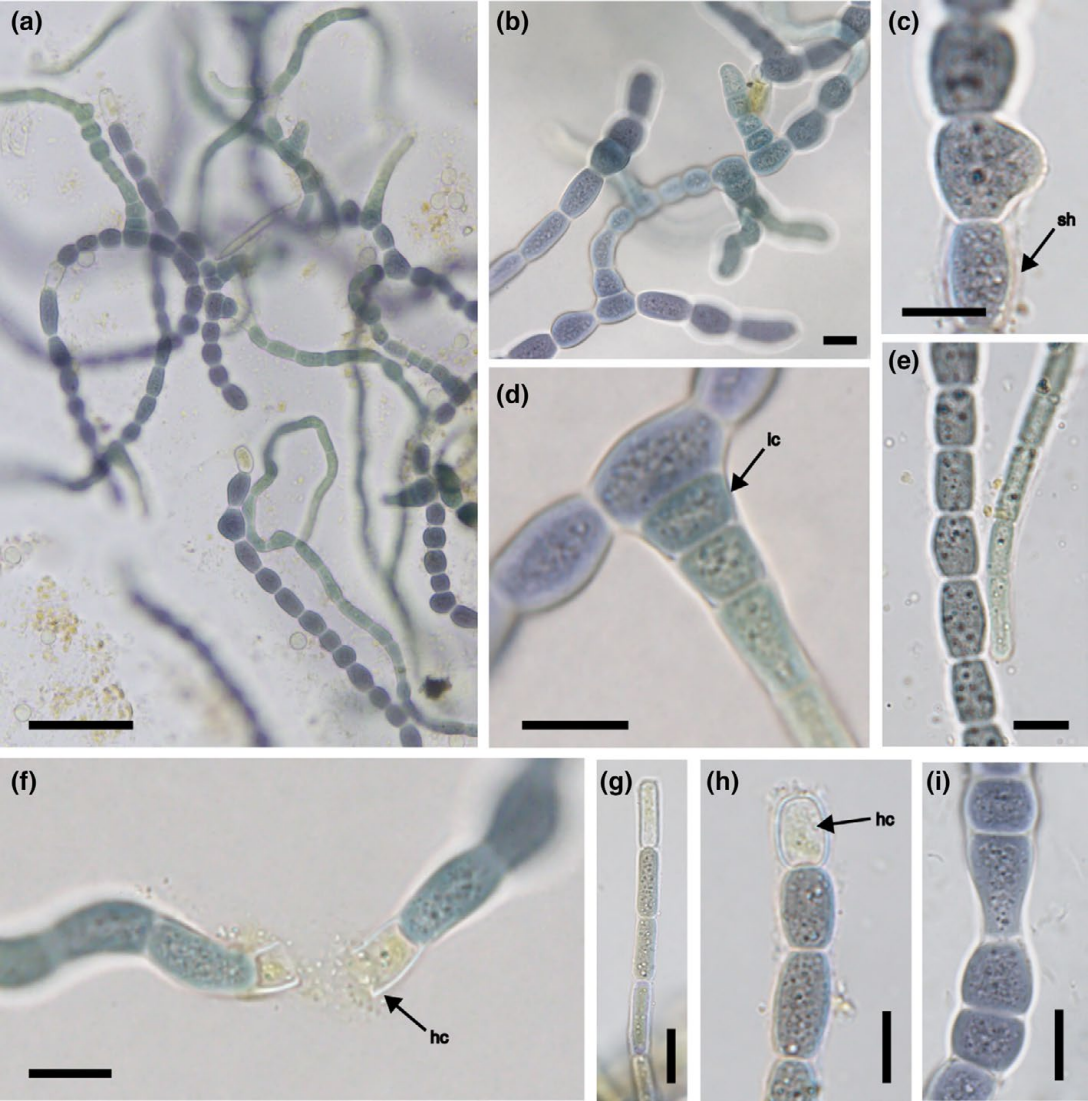
The overall morphology of *Mastigocladus boudae*, strain Alb13‐JP1. Light microscopy micrographs: entwined filaments (a, b), initial phase of branch formation (c), mature branch with adherent initial cell (d), comparison of main and branch parts of filament (e), main filament with broken intercalary heterocyte (f), seemingly terminal heterocyte on branch (g) and main filament (h), stretched cell of the main filament (i). sh—sheath, ic—initial cell, hc—heterocyte. Scale bars 50 μm (a); 10 μm (b–i).

**Diagnosis:** Only solitary intercalary heterocytes are present, cells of the branches are very long, to 14 μm, initial cells are wider than long. Some main filament cells are characteristically stretched. Phylogenetically, it is distant from the other species of the genus. *Mastigocladus boudae* is characterized by its unique sequences (GenBank accession numbers PQ651698 Alb13‐JP1 clone 4b, operon without tRNA and PQ651696 Alb13‐JP1 clone 1b operon with tRNA^Ala^ and tRNA^Ile^) for the 16S rRNA gene and 16S–23S ITS rRNA region, and its phylogenetic position.

**Description:** Colony dark blue green, trichomes blue‐green, or slightly violet. Trichomes straight or irregularly coiled, with thin (almost invisible) colorless sheaths. True branching is frequent, T‐type, multilateral; at some parts of the thallus, unilateral branching prevails. Main filament with barrel‐shaped cells, approximately isodiametric, constricted at cross‐walls, 8–12 μm wide (10.1 ± 2.1) and 8–15 μm long (11.55 ± 3.55). Some main axis cells have one side stretched out, forming a concave strait. Lateral branches usually continually tapering at the ends. Cells are cylindrical, usually longer than wide (especially young branches), 5–10 μm wide (7.65 ± 2.45) and 6–14 μm long (10.15 ± 3.95). Initial cells of the branches are wider than long, 8.3–10.5 μm wide (9.4 ± 1.1) and 5.1–8 μm long (6.6 ± 1.5). Terminal cells are rounded or slightly tapering, without calyptra. Heterocytes are intercalary, solitary, mostly cylindrical, of the same size or slightly larger than vegetative cells. The filament often breaks at the heterocyte pore; therefore, some heterocytes are positioned seemingly terminally. Akinetes are not observed; reproduction is by few‐celled hormogonia.

**Holotype:** CBFS A‐263, dried material of the reference strain CCALA 10346 preserved on paper in a metabolically inactive state, kept at CBFS, *leg*. M. Wipplingerová and J. Kaštovský, 1 Nov. 2022, originally growing on a periodically wetted area above the surface of the underground lake in the cave entrance

**Type locality:** Entrance to Sulfur Cave, Vromoner Canyon, Greece/Albania border (40°5′46.539″ N, 20°40′43.841″ E)

**Ecology:** Microscopic colony just above the surface of the underground lake in the cave entrance area (probably often as underwater locality), water with high content of hydrogen sulfide, illuminance between 5 and 15 lx. It has not been observed in natural samples, only after laboratory cultivation, probably a rare species.

**Etymology:** *Boudae*—named to honor Richard Bouda, speleologist, who co‐organized this expedition

**Reference strain:** CCALA 10346 (housed at Culture Collection of Autotrophic Organisms at the Institute of Botany, Třeboň, CZ), isolated as Alb13‐JP1 by J. Pokorný

#### *Pegethrix sulphurea* J.Pokorný & Kaštovský sp. nov. (Figure 4)

**FIGURE 4:**
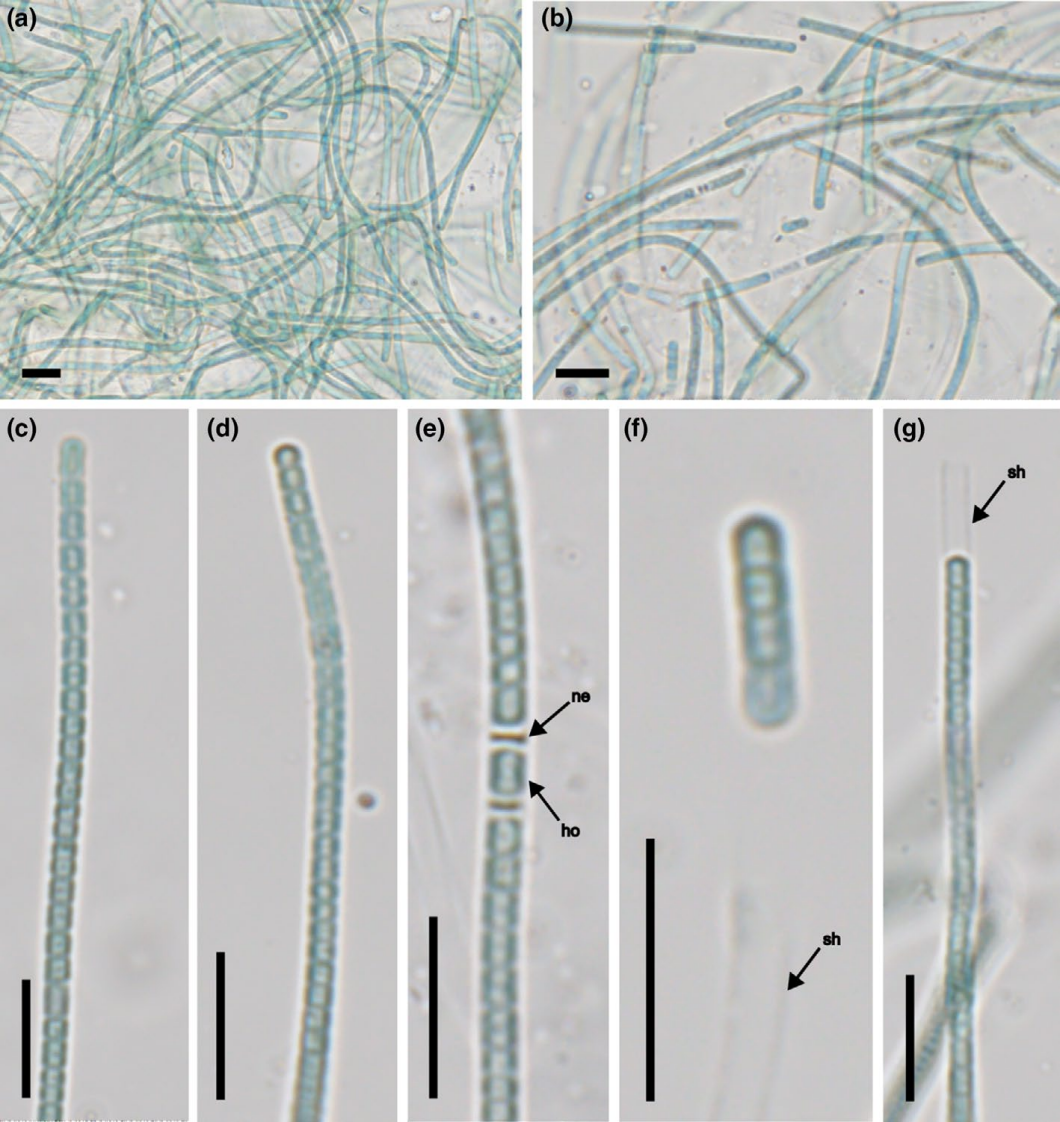
The overall morphology of *Pegethrix sulphurea*, strain Alb13‐JP2, light microscopy micrographs. Entwined filaments (a, b), detail on individual filaments (c, d), detail on necridia with hormogonium forming (e), detail on hormogonium (f), sheathed filament (g). sh—sheath, ne—necridium, ho—hormogonium. Scale bars 10 μm (a–g).

**Diagnosis:** Cryptic species from the other species of *Pegethrix* differs mainly by the phylogenetic distance and the different secondary structures of the 16S–23S ITS rRNA region (GenBank accession number PQ651699 of strain Alb13‐JP2 clone 1). It is the only so far described species of the genus growing in thermal sulfur springs.

**Description:** Thallus light blue‐green, fine mat, radially spreading on agar, filaments dark blue‐green to gray‐blue‐green. Trichomes long, many‐celled, straight to heavily bent or wavy, and entwined, without nodules or knots, with necridia, not tapered. Sheaths present in older filaments, firm, thin, colorless, usually overlapping the trichome or present without any cells inside, completely absent in all loose hormogonia. Cells cylindrical, strongly constricted at cross‐walls, probably with parietal thylakoids. Cells mainly shorter than wide, rarely isodiametric, occasionally longer than wide shortly prior to division, 1.8–2.2 μm wide (2 ± 0.2) and 0.8–2.3 μm long (1.55 ± 0.75). Filaments up to 3.2 μm wide. The division within the trichome is irregular without apparent meristematic zones. Apical cells cylindrical with rounded ends to spherical, rarely conical, with identical dimensions as the rest of the cells in the trichome. Reproduction occurs through hormogonia of variable length.

**Holotype:** CBFS A‐262, dried material of the reference strain CCALA 10345 preserved on paper in a metabolically inactive state, kept at CBFS, *leg*. M. Wipplingerová and J. Kaštovský, 1 Nov. 2022, originally growing on a periodically wetted area above the surface of the underground lake in the cave entrance

**Type locality:** Entrance to Sulfur Cave, Vromoner Canyon, Greece/Albania border (40°5′46.539″ N, 20°40′43.841″ E).

**Ecology:** Microscopic colony just above the surface of the underground lake in cave entrance area (probably often as underwater locality), water with high content of hydrogen sulfide, illuminance between 5 and 15 lx

**Etymology:** *Sulphurea*—named to highlight its presence in an environment rich in sulfur

**Reference strain:** CCALA 10345 (housed at Culture Collection of Autotrophic Organisms at the Institute of Botany, Třeboň, CZ), isolated as Alb13‐JP2 by J. Pokorný

**FIGURE 5:**
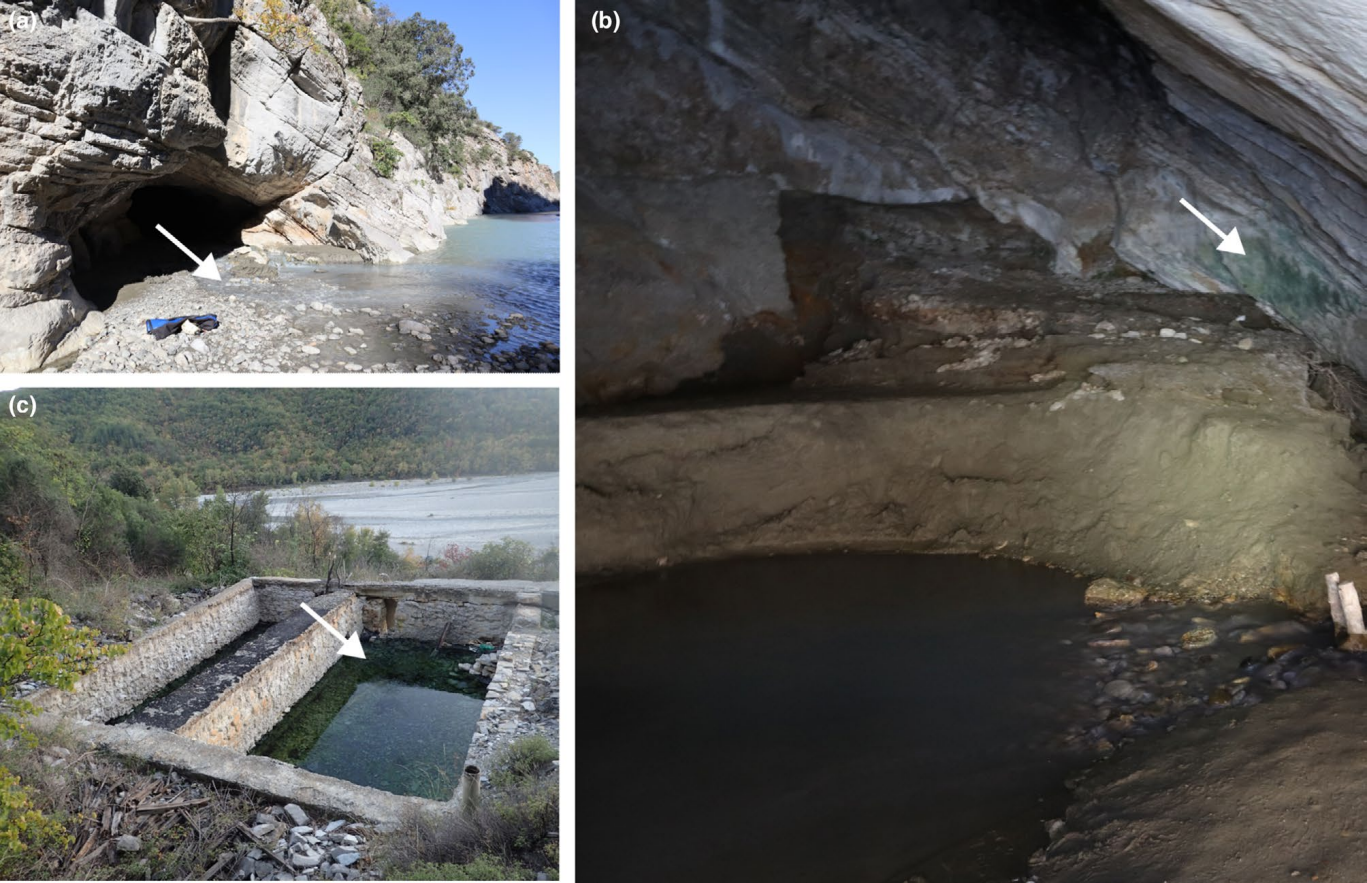
The type localities of herein circumscribed new taxa. (a) entrance to the cave Sulfur (sample Alb13, type locality of *Mastigocladus boudae* and *Pegethrix sulphurea*), (b) wall inside Sulfur cave (sample Alb16, type locality of *Loriellopsis vromonerensis*), (c) spa Xomos (sample Alb61 and Alb62, type locality of *Xomosiella audyi*). White arrows highlight approximate sampling points.

All type localities are shown in Figure 5.

### Molecular and phylogenetic analyses

#### Xomosiella

**FIGURE 6:**
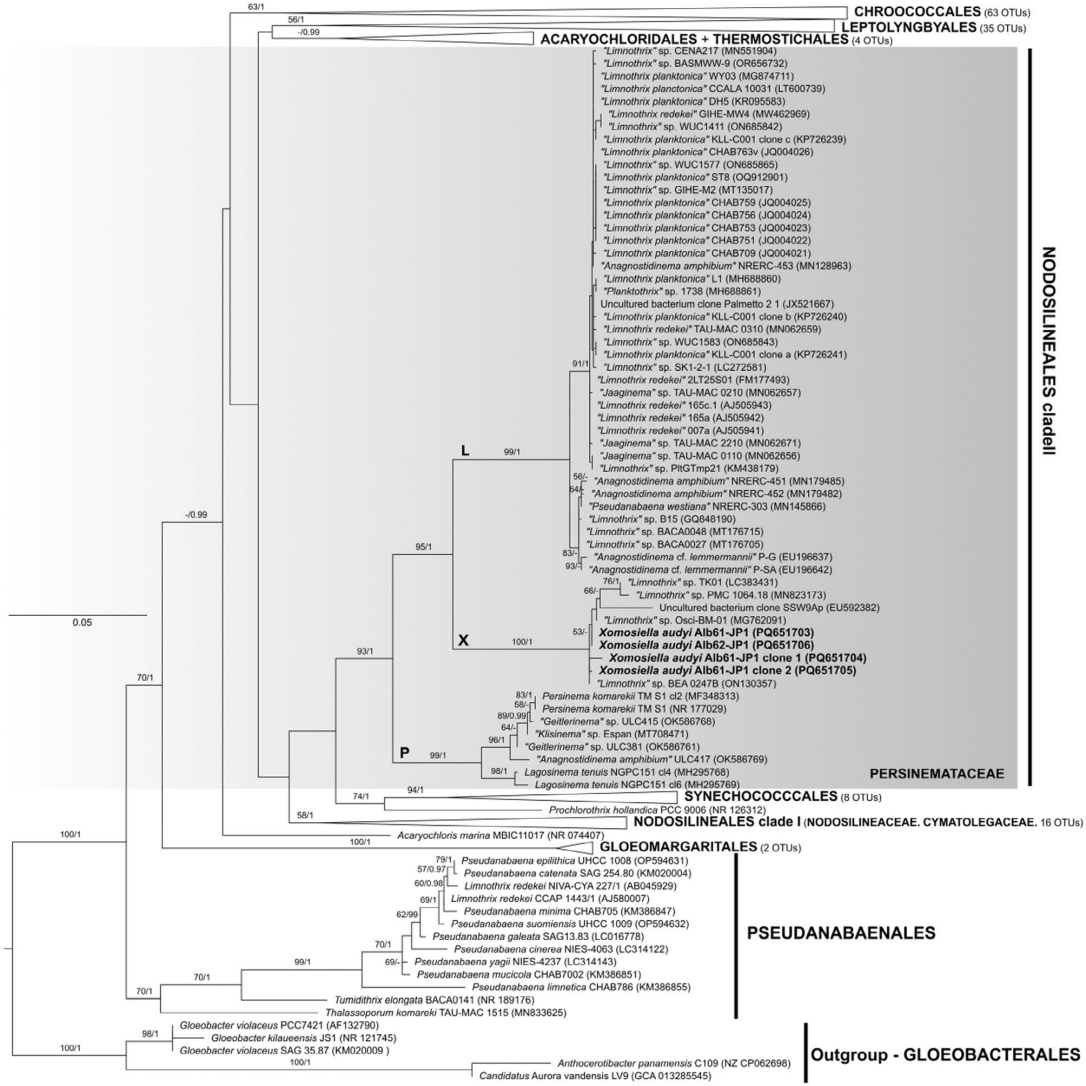
The phylogenetic tree constructed from the 16S rRNA gene sequences of 203 taxa, 1096 positions long, showing the phylogenetic position of *Xomosiella* in Persinemataceae (Nodosilineales) with five sequences of Gloeobacterales applied as an outgroup. Representatives of Chroococcales, Leptolyngbyales, Acaryochloridales, Thermostichales, Synechococcales, Gloeomargaritales, and Pseudanabaenales were also included in the analyses. The topology represents the best ML tree with the best model GTR + R chosen by SMS. Node support includes the bootstrap of ML analysis above 50 and posterior probabilities of the BI analysis above 0.95. Our four sequences of *X*. *audyi* are highlighted in bold. The full uncollapsed tree is available in Figure S1.

The sequences of two strains of *Xomosiella* (Alb61‐JP1 and Alb61‐JP2) were placed in a single well‐supported clade (clade X) sister to a sequence cluster containing operational taxonomic units (OTUs) designated as *Limnothrix*, *Planktothrix*, *Anagnostidinema*, and *Jaaginema* (clade L); however, this clade did not include reference strains of either taxon (Figure 6). Sister to these clades was a true lineage of the family Persinemataceae (Nodosilineales), *Lagosinema* and *Persinema* (P) represented by their respective reference strains (Akagha et al., 2019; Heidari et al., 2018). Sequences within the X clade shared high 16S rRNA gene similarity (PD 0%–2.26%) and were significantly divergent from the neighboring taxa (PD > 6.99%), thus belonging to a new genus phylogenetically isolated from the other Persinemataceae (Table 1). This genus, so far, contains sequences from various locations and habitats (see the Discussion for detail). All sequences of the two strains of *Xomosiella audyi*, Alb61‐JP1 and Alb62‐JP1, at both the 16S rRNA gene (PD = 0.60%) and ITS rRNA region (PD = 2.00%) level, were members of a single species. Furthermore, the secondary structures of the ITS rRNA region semi‐conserved domains were identical among our sequences of *X. audyi*. The sequences of *Limnothrix* spp. PMC 1064.18 and TK01 were more divergent from *X. audyi* (0.93%–1.55%). The only taxon without epithet in this clade, uncultured bacterium clone SSW9Ap from Salton Sea, California (Dillon et al., 2009), differed from the *X. audyi* 16S rRNA gene sequences by 1.73%–2.36% and possibly represents yet another species within the herein described genus. The taxa belonging to all three clades (P, X, L) were phenotypically very similar, with simple trichomes, cells longer than wide with similar dimensions, facultatively bearing granules (Akagha et al., 2019; Gkelis et al., 2005; Heidari et al., 2018; Perkerson III et al., 2010; Zhu et al., 2012); thus, the diversity was mainly cryptic. In our analyses (Figures 6 and S1) the order Nodosilineales did not cluster monophyletically, whereas the family Persinemataceae (clade II) was isolated from the families Nodosilineaceae and Cymatolegacae, which did cluster as sister taxa (clade I).

**TABLE 1:** Range in percent 16S rRNA gene sequence dissimilarity (p‐distance) of *Xomosiella audyi* and related strains in Persinemataceae, based on a distance of all OTUs within a clade to all OTUs in the comparison clade.

		1	2	3	4	5
1	*Xomosiella audyi*					
2	*Xomosiella—*other strains	0.00–2.26				
3	“*Limnothrix*” clade L	6.99–8.32	6.59–9.01			
4	*Persinema*	7.14–7.93	6.72–8.30	7.66–8.86		
5	*Lagosinema*	8.12–8.39	7.55–8.40	7.03–7.76	2.37–3.01	

*Note*: Full table is available in the Table S1.

**FIGURE 7:**
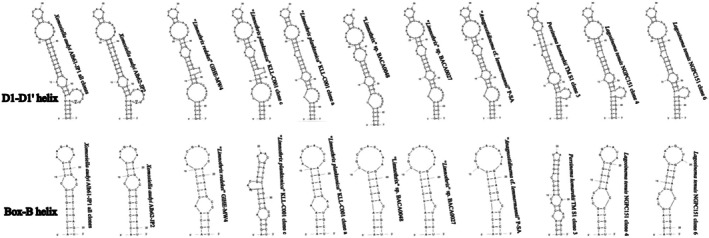
D1‐D1′ and Box‐B helices from representative Persinemataceae sequences.

**FIGURE 8:**
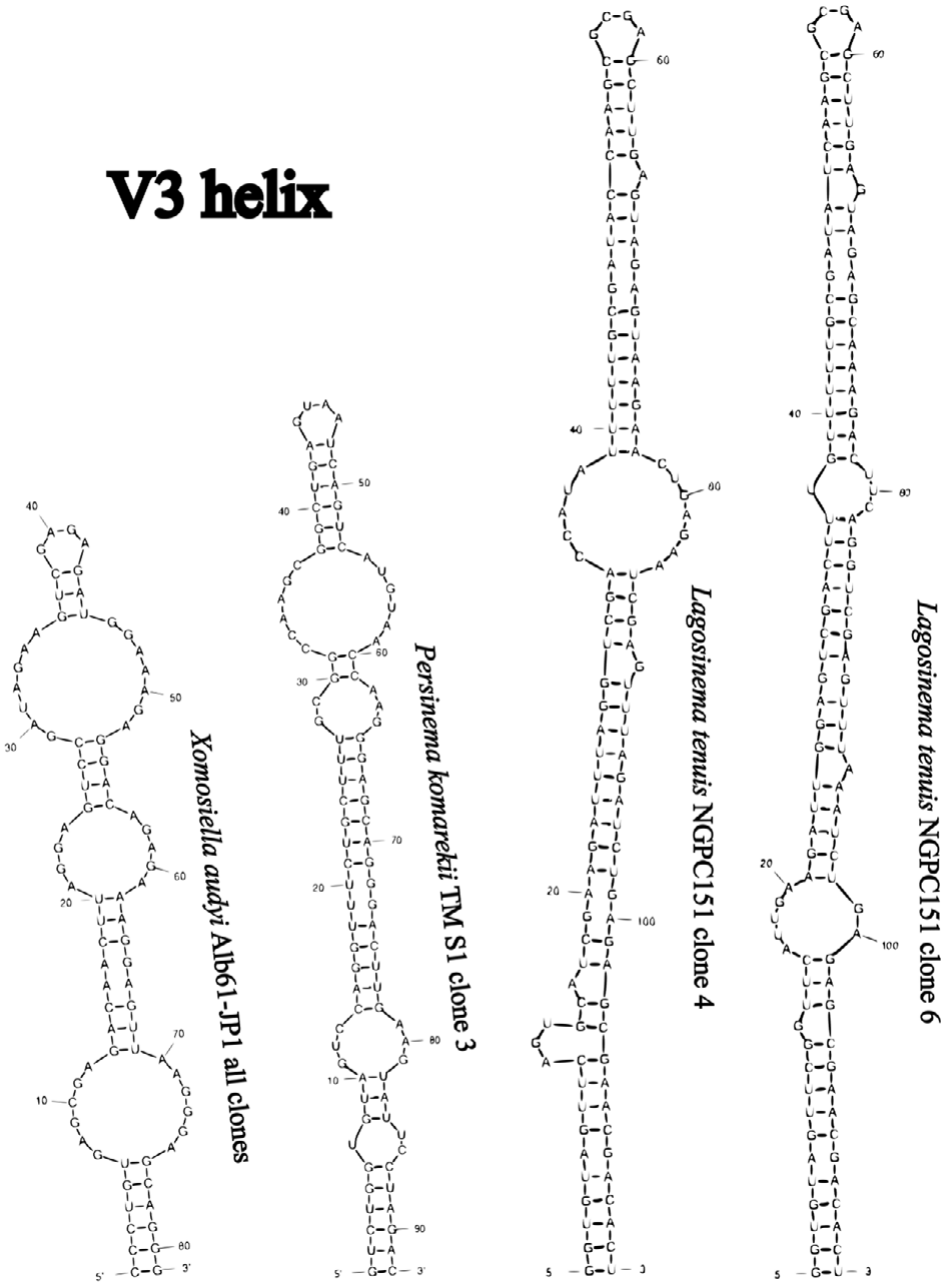
Available folded V3 helices from representative Persinematacae taxa.

None of the sequences belonging to clade L contained the entire ITS rRNA region, and therefore, we were unable to compare the V3 structures with confidence. Several representative sequences were chosen to infer the structures of D1‐D1′ and Box‐B helices for illustration (Figure 7). All investigated sequences of Persinemataceae contained one tRNA^Ile^. The secondary structures of the D1‐D1′ helix among the investigated sequences of the X, L, and P clades were conservative in length; however, they differed in the length of the basal 3′‐end unilateral bulge and presence of unpaired nucleotides at the 5′‐end opposite to it, as well as in the pairing and presence of additional bulges or residual nucleotides within the stem of the helix. Box‐B in strains Alb61‐JP1 and Alb62‐JP1 did not have the typical AGCA–TGCT clamp; instead, they consistently read AGCG–CGCT. However, the typical clamps were present in all the closest investigated related taxa outside the putative new genus; therefore, this mutation may be a trait specific to the herein described genus *Xomosiella*. The V3 helix of *X. audyi* was much shorter than that of *Lagosinema tenuis* and *Persinema komarekii* and differed in the number and position of bulges (Figure 8). The full structure was recovered only from the strain Alb61‐JP1.

#### Loriellopsis

**FIGURE 9:**
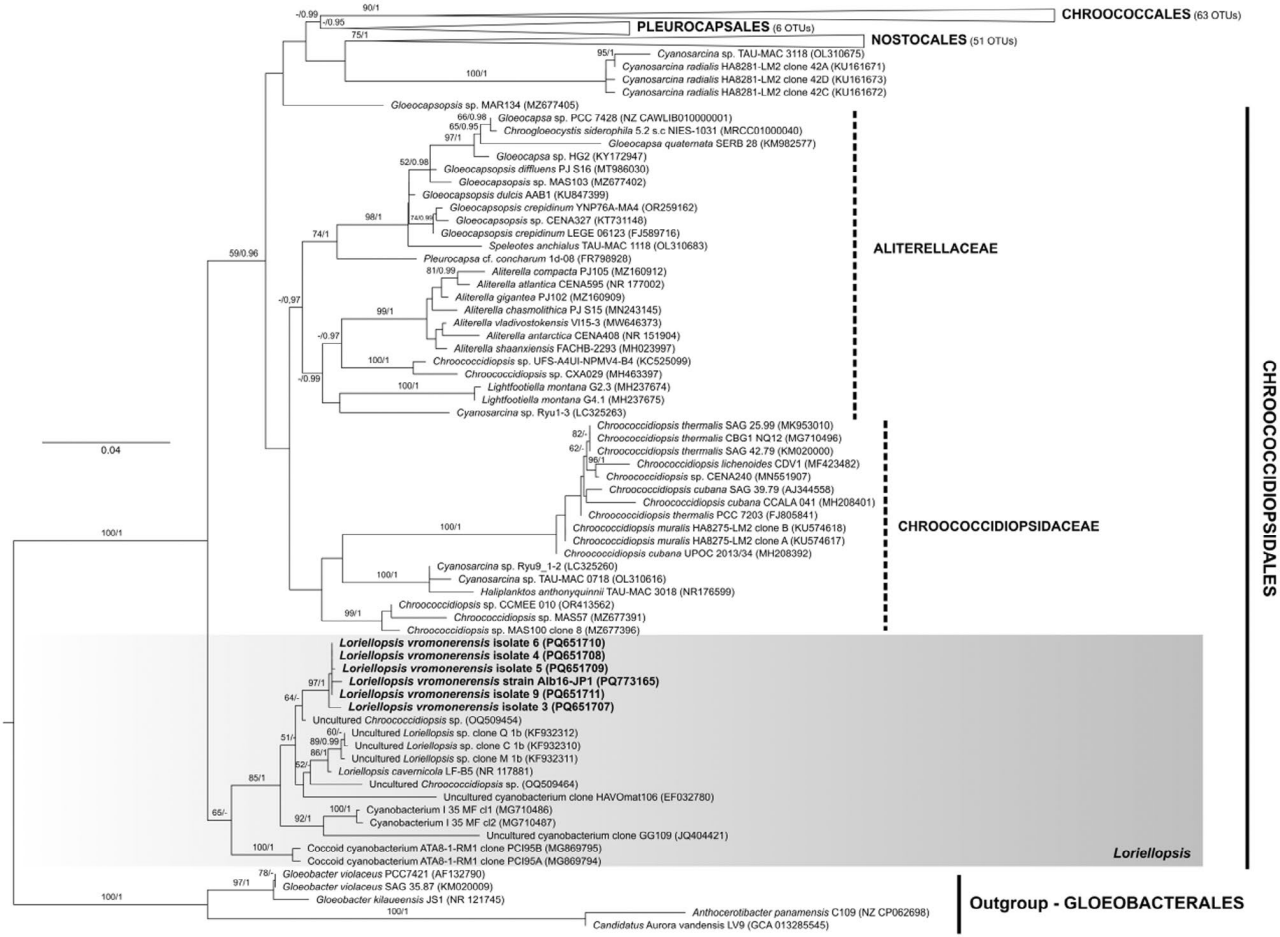
The phylogenetic tree constructed from the 16S rRNA gene sequences of 189 taxa, 1096 positions long, showing the phylogenetic position of *Loriellopsis vromonerensis* with five sequences of Gloeobacterales applied as an outgroup. Representatives of Chroococcales, Pleurocapsales, and Nostocales were also included in the analyses. The topology represents the best ML tree with the best model GTR + R chosen by SMS. Node support includes the bootstrap of ML analysis above 50 and posterior probabilities of the BI analysis above 0.95. Our six sequences of *Loriellopsis vromonerensis* are highlighted in bold. The full uncollapsed tree is available in the Figure S2.

The five successfully sequenced isolates of the coccal sarcinoid cyanobacterium from sample Alb‐16 and the strain Alb16‐JP1 formed a well‐supported monophyletic cluster within a larger group containing taxa with morphologically very heterogeneous thalli. A significant fraction of this group was composed of uncultured coccal cyanobacteria morphologically similar to *Cyanosarcina/Chroococcidiopsis*, but this cluster also contained true branching heterocystous cyanobacteria of the genus *Loriellopsis* (Symphyonemetaceae, Nostocales), including its type strain (Lamprinou et al., 2011; Figure 9). Our isolates were genetically congruent (16S rRNA gene PD < 0.47%) and shared close similarity to this *Loriellopsis* reference strain (PD = 1.83%–2.45%, Tables 2 and S2). We have described the taxonomic problems with this strain in the Discussion; briefly, we consider this *Loriellopsis* reference sequence to be mis‐determined. Morphologically, our isolates closely resemble the genus *Chroococcidiopsis* or *Cyanosarcina* due to the polyhedral cells arranged in irregular colonies with thin common mucilage (Komárek & Anagnostidis, 1999; Kováčik, 1988; Miscoe et al., 2016). However, such taxa clustered outside this clade, and the order Chroococcidiopsidales was paraphyletic in our analyses, with the position of most of its genera uncertain. Because of their considerable phylogenetic distance from any extant genus, we have defined this cluster as a new genus with a sarcinoid organization of coccal‐celled colonies.

**TABLE 2:** Range in percent 16S rRNA gene sequence dissimilarity (p‐distance) of *Loriellopsis vromonerensis* and related strains, based on a distance of all OTUs within a clade to all OTUs in the comparison clade.

		1	2	3	4	5	6	7
1	*Loriellopsis vromonerensis*							
2	*Loriellopsis cavernicola*	1.76–2.45						
3	*Loriellopsis* genus other strains	0.88–6.04	1.36–5.84					
4	*Chroococcidiopsis*	7.66–9.64	7.57–9.18	7.40–10.19				
5	*Haliplanktos*	6.82–8.48	6.72–8.26	6.72–9.31	6.45–8.72			
6	“*Chroococcidiopsis*” undescribed taxon	6.16–7,26	5.94–6.88	5.84–8.67	6.39–8.47	4.24–6.08		
7	*Gloeocapsopsis* sp. MAR134 (MZ677405)	5.84–6.33	6.24–6.59	5.67–7.49	7.21–8.36	5.63–6.64	4.57–5.11	

*Note*: Full table is available in the Table S2.

**FIGURE 10:**
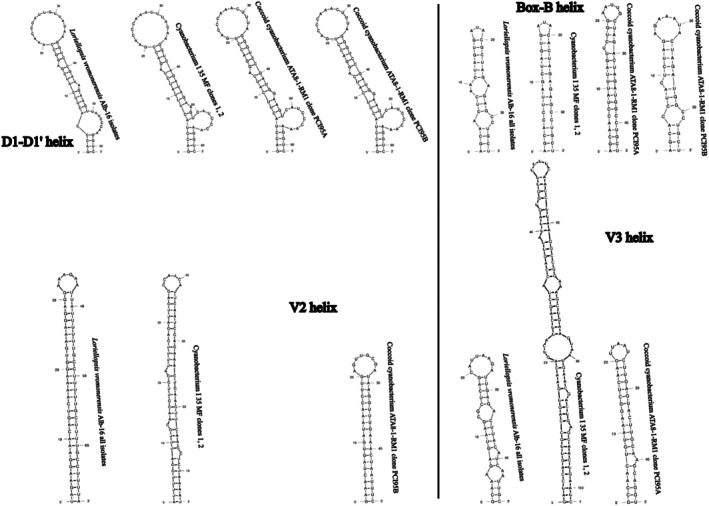
Structures of D1‐D1′, Box‐B and V3 helices from representative taxa from immediate neighborhood of *Loriellopsis*.

All *Loriellopsis vromonerensis* sequences contained both tRNAs. The only taxa from their close phylogenetic proximity with ITS rRNA region sequences available were clones of cyanobacterium I 35 MF and ATA8‐1‐RM1 (Figure 10). Interestingly, two of three clones of ATA8‐1‐RM1 (PCI95A and PCI95D) did not contain any tRNAs, and one contained both (PCI95B), suggesting the presence of two operon variants in the entire clade but underestimated due to insufficient sampling. The D1‐D1′ structures in this clade were all the same length, differing only by minor details in folding patterns. The V2 structure did not form any significant bulges in any taxon but differed significantly in ATA8‐1‐RM1 in that it was much shorter than the rest. The Box‐B was rather short in all taxa, forming two short bilateral or unilateral bulges and differing slightly in pairing patterns and length of the terminal loop. The V3 helix was very long in cyanobacterium I 35 MF (102 nt compared to 47 nt in *L*. *vromonerensis* and 46 nt in cyanobacterium ATA8‐1‐RM1).

#### Mastigocladus

**FIGURE 11:**
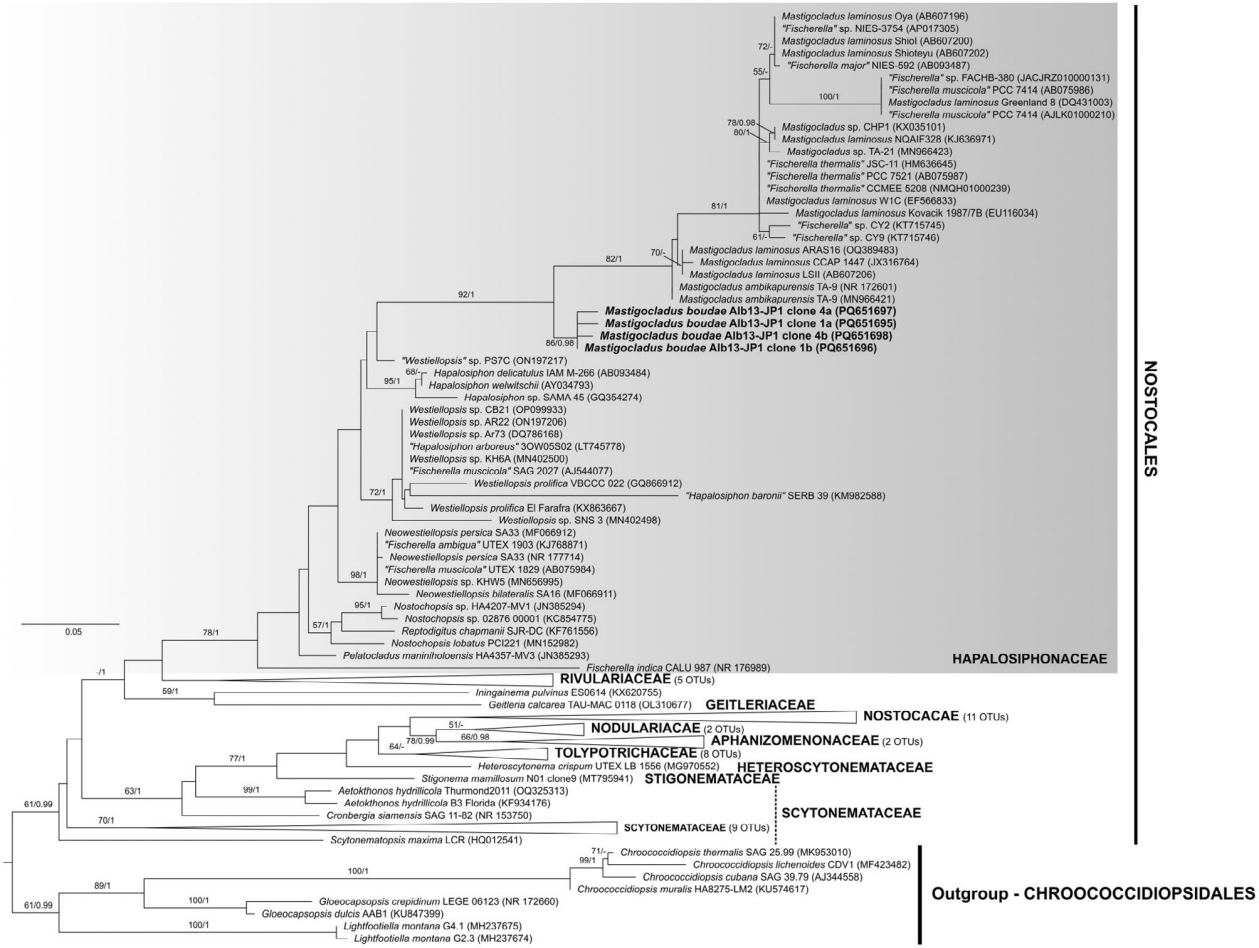
The phylogenetic tree constructed from the 16S rRNA gene sequences of 106 taxa, 1096 positions long, showing the phylogenetic position of *Mastigocladus boudae* in Hapalosiphonaceae with eight sequences of Chroococcidiopsidales applied as an outgroup. Other representatives of Nostocales were also included in the analyses. The topology represents the best ML tree with the best model GTR + R chosen by SMS. Node support includes the bootstrap of ML analysis above 50 and posterior probabilities of the BI analysis above 0.95. Our four sequences of *M. boudae* are highlighted in bold. The full uncollapsed tree is available in Figure S3.

The four cloned sequences of the strain Alb13‐JP1 clustered congruently inside a single clade with significant support, forming a monophyletic group with *Mastigocladus* and *Fischerella* sequences in Hapalosiphonaceae (Nostocales; Figure 11). Based on rules expressed for this family by Casamatta et al. (2020) following Johansen and Casamatta (2005) and Yarza et al. (2014), this strain belonged to a new species of the genus *Mastigocladus*, herein proposed as *Mastigocladus boudae*. The cloned 16S rRNA gene sequences of Alb13‐JP1 were monophyletic and 0.28%–0.74% dissimilar to each other. The clade was divergent on the 16S rRNA gene level from its closest relatives, with the lowest dissimilarity (PD = 2.32%) to *Mastigocladus ambikapurensis* (Jaiswal et al., 2021). However, the highest dissimilarity from any other OTU within the *Mastigocladus* clade was PD < 5.41% (“*Fischerella muscicola*” PCC 7414, AB075986; Tables 3 and S3), and thus, the establishment of a new genus would be obsolete. The entire family Hapalosiphonaceae was monophyletic in our analyses, but with poor support for the positions of the genera within. The morphology of the strain Alb13‐JP1 was in agreement with the general description of *Mastigocladus*, with true branching, uniseriate trichomes, narrow branches with extended cells, and irregularly long main filament cells (Kirchner, 1898; Komárek, 2013).

**TABLE 3:** Range in percent 16S rRNA gene sequence dissimilarity (p‐distance) of *Mastigocladus boudae* and related strains in Hapalosiphonaceae, based on a distance of all OTUs within a clade to all OTUs in the comparison clade.

		1	2	3	4	5	6	7	8	9
1	*Mastigocladus boudae*									
2	*Mastigocladus laminosus*	3.43–5.41								
3	*Mastigocladus ambikapurensis*	2.32–3.19	1.46–3.26							
4	*Hapalosiphon*	3.71–4.36	4.11–6.39	4.20–4.92						
5	*Westiellopsis*	3.65–8.00	4.11–9.07	4.84–8.62	1.07–5.96					
6	*Neowestiellopsis*	3.43–4.45	4.56–6.72	4.93–5.82	1.41–2.57	1.41–6.14				
7	*Reptodigitus* + *Nostochopsis*	3.90–5.10	4.21–5.42	4.49–5.82	1.74–3.24	1.37–6,78	1.63–2.65			
8	*Pelatocladus*	3.45–3.82	4.13–5.23	4.22–4.50	1.74–2.30	2.02–6.18	1.84–2.39	1.47–2.20		
9	*Fischerella indica* CALU 987	6.52–6‐92	7.09–8.24	6.90–7.39	4.76–6.03	5.54–8.76	5.15–5.75	5.46–6.22	5.08	

*Note*: Full table is available in the Table S3.

**FIGURE 12:**
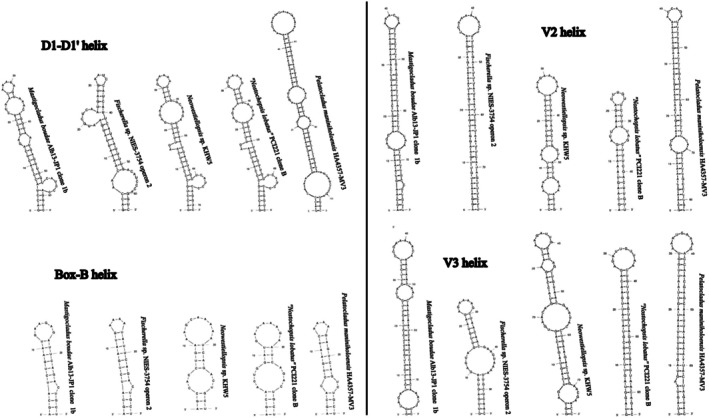
Structures of D1‐D1′, V2, Box‐B, and V3 helices from operons including both tRNA^Ile^ and tRNA^Ala^ of representative Hapalosiphonaceae taxa.

**FIGURE 13:**
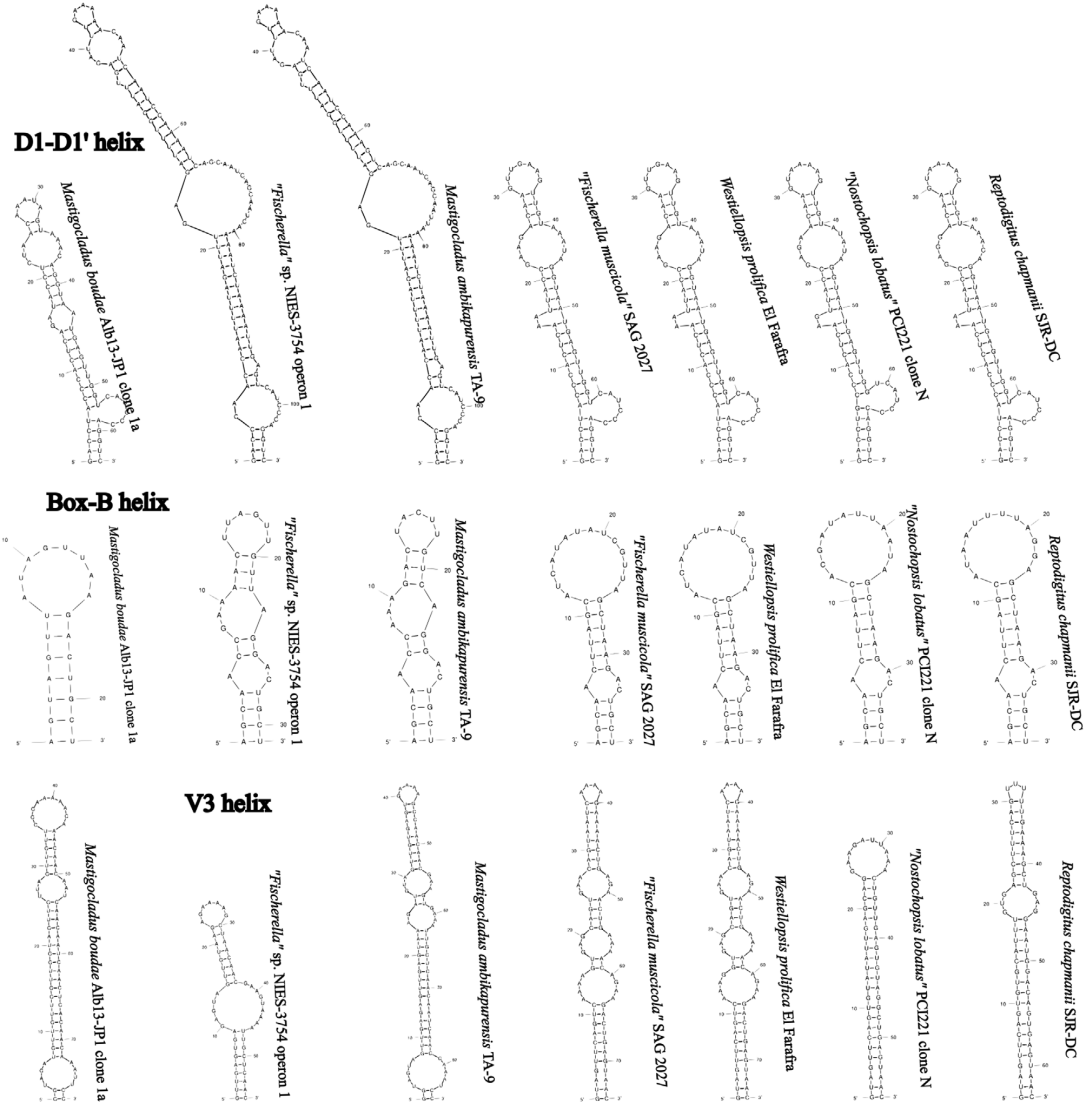
Structures of D1‐D1′, Box‐B, and V3 helices from representative Hapalosiphonaceae taxa, operons lacking tRNA.

A single cloned ITS rRNA region sequence (Clone 1b) contained both tRNA^Ala^ and tRNA^Ile^, and the ITS rRNA region sequences containing no tRNA (Clones 1a, 4a, and 4b) shared high similarity (PD < 1.20%). Of the rest of Hapalosiphonaceae, operons with both tRNAs were recovered from *Mastigocladus* (“*Fischerella*” sp. NIES‐3754), *Neowestiellopsis*, *Nostochopsis lobatus*, and *Pelatocladus* (Figure 12). The secondary structures of the D1‐D1′ and V3 helices of both ITS rRNA region variants were identical; however, the Box‐B structure was 29 nt long in the variant with both tRNAs; it was only 22 nt in the variant missing tRNA, which also had different bases and a 3′–end single cytosine residue. The D1‐D1′ helix was much shorter than the rest of the *Mastigocladus* clade, and it formed a unilateral and not bilateral basal bulge. The mid‐helix bulge was also much shorter. It was rather similar in length and shape to the rest of the Hapalosiphonaceae (Figure 13). The V2 helix of the tRNA‐bearing operon differed greatly in the total length, number, length, and position of the bulges. The Box‐B helices were mostly conserved, differing in *Mastigocladus* by mid‐helix pairing. The V3 helices differed similarly to V2 in the overall folding pattern.

#### Pegethrix

**FIGURE 14:**
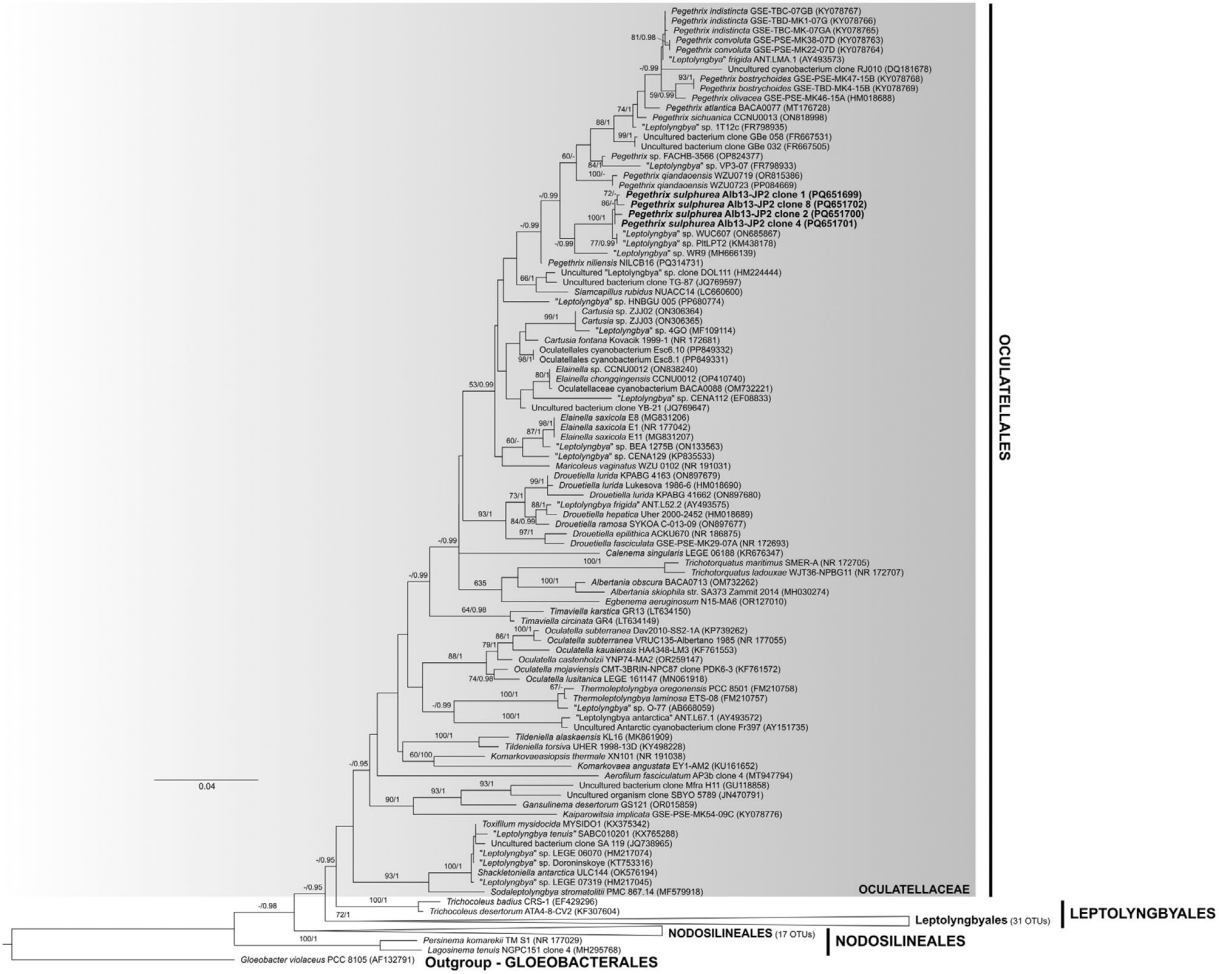
The phylogenetic tree constructed from the 16S rRNA gene sequences of 145 taxa, 1157 positions long, showing the phylogenetic position of *Pegethrix sulphurea* within Oculatellaceae with one sequence of Gloeobacterales applied as an outgroup. Representatives of Nodosilineales and Leptolyngbyales were also included in the analyses. The topology represents the best ML tree with the best model GTR + R chosen by SMS. Node support includes the bootstrap of maximum likelihood analysis above 50 and posterior probabilities of the Bayesian Inference analysis above 0.95. Our four sequences of *P*. *sulphurea* are highlighted in bold. The full uncollapsed tree is available in Figure S4.

The four cloned sequences of our strain of a simple trichal cyanobacterium clustered in a monophyletic clade within the genus *Pegethrix* (Oculatellales, Oculatellaceae). Although the position of our strain was greatly supported only in the Bayesian analysis, the topology of the *Pegethrix* clade in the ML analysis was identical (Figure 14). Furthermore, the 16S rRNA gene PD of our strain to all other *Pegethrix* species was between 2.51% and 4.78% (Tables S4 and S5), and the general morphology also corresponded to the characteristics of this genus (Mai et al., 2018). Thus, our isolate represented a new distinct, separate species of *Pegethrix*, herein described as *Pegethrix sulphurea*. Two strains of *Leptolyngbya*‐like cyanobacteria (WUC607, PltLPT2) also belonged to this species according to the tree topology and 16S rRNA gene PD < 0.55%.

**FIGURE 15:**
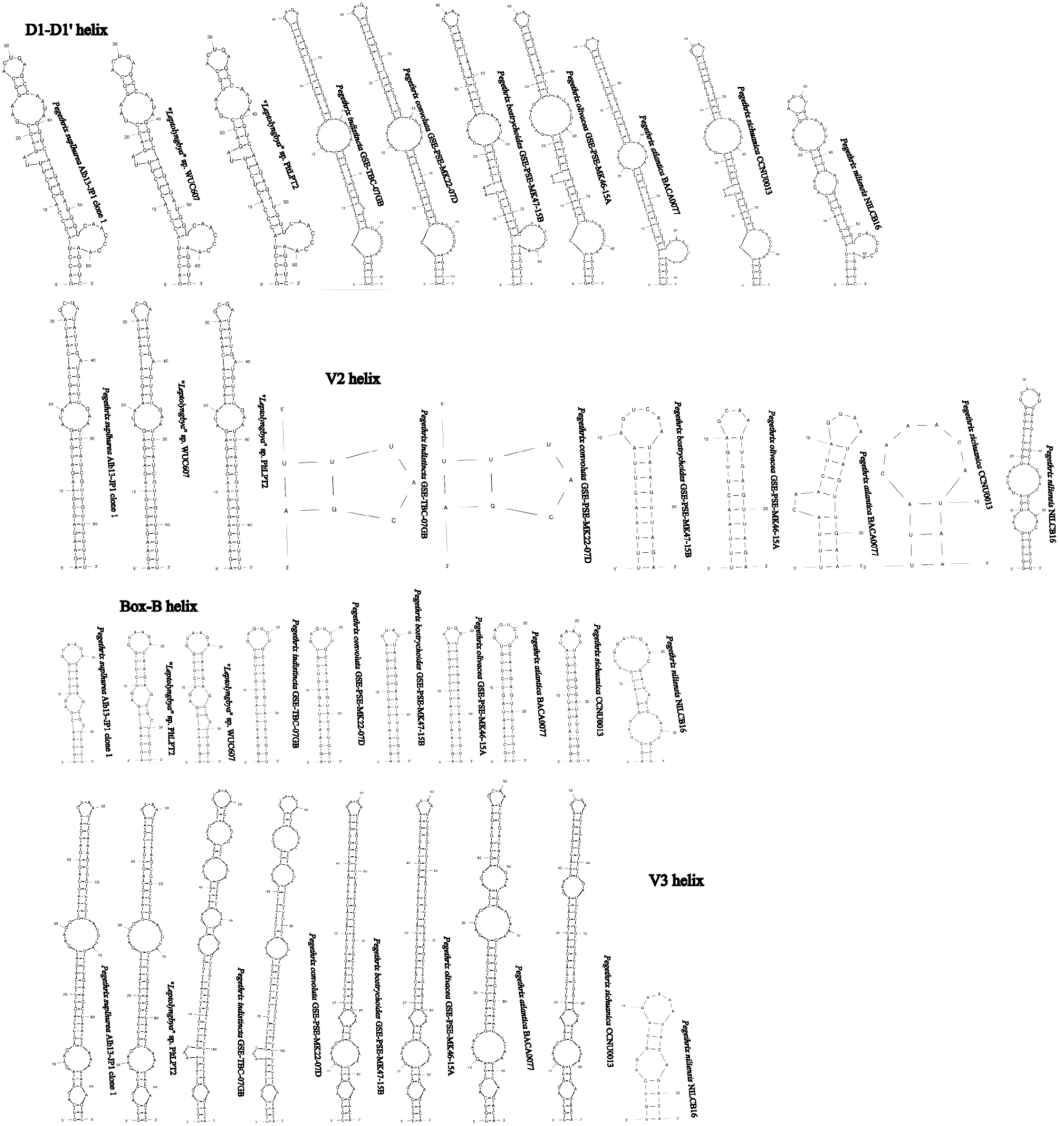
Structures of D1‐D1′, V2, Box‐B, and V3 helices from all available *Pegethrix* species.

All *Pegethrix* 16S–23S ITS rRNA region sequences contained tRNA^Ala^ and tRNA^Ile^, and all four analyzed semi‐conserved domains were folded. These helices of *P*. *suphurea* were identical to those of “*Leptolyngbya*” sp. PltLPT2 and WUC607 except for the structure of the V3 helix (missing in WUC607), which further supported their belonging to the same species. As reported by other authors (Hentschke et al., 2024; Luz et al., 2023; Mai et al., 2018; Shen et al., 2023), the helical structures across the genus *Pegethrix* were highly variable in both length and shape. *Pegethrix sulphurea* was distinct by its relatively short D1‐D1′ helix, comparable only to *P*. *niliensis* but differing by the mid‐helix pairing and terminal loop sequence. The sequence 5′– CAUCCCA −3′ in the basal unilateral bulge typical for *Pegethrix* was also replaced by 5′– CAACCCA −3′. Conversely, its V2 helix was exceptionally long, spanning 65 nt, whereas the second longest belonged to *P*. *niliensis* (58 nt) and the shortest to *P*. *convoluta* and *P*. *indistincta* with one bilateral bulge and a 3′– end adenine residue. The Box‐B helix differed from the other species by one short bilateral bulge and a 3′– end cytosine residue. The V3 differed greatly in the pairing, number, and position of mid‐helix bulges (Figure 15).

**FIGURE 16:**
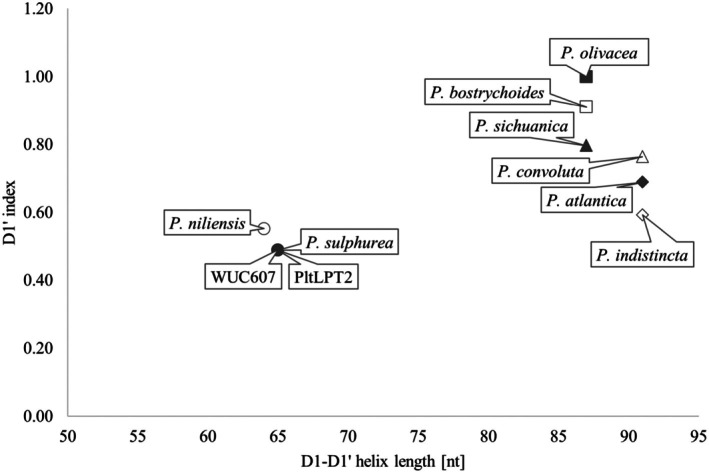
DIV plotted against the total length of the D1‐D1′ helices of the available species of Pegethrix.

Furthermore, the D1′ index values plotted against the total length of the D1‐D1′ helix showed probable divergence of two orthologous groups of ITS rRNA region operons. One group consisted of *Pegethrix niliensis* and *P*. *sulphurea*, and the other encompassed the rest of the known *Pegethrix* species. This followed the phylogenetic results that predict *P*. *niliensis* and *P*. *sulphurea* as the closest relatives, with the remaining species being sister to *P*. *sulphurea* (Figure 16).

## DISCUSSION

### Xomosiella

*Xomosiella* was a clearly separated genus within Persinemataceae (16S rRNA gene PD > 6.59% compared with any other genus), although the morphology was very similar to the so‐far described genera of this family, *Lagosinema* and *Persinema* (clade P), because all are thin, unbranched filamentous species with prominent granules within cells (Akagha et al., 2019; Heidari et al., 2018). Additionally, an enigmatic genus containing mostly sequences of “*Limnothrix*” was sister to *Xomosiella*. The genus *Xomosiella*, so far, contains the isolates from Albania presented here and those of “*Limnothrix*” spp. originating from Guadeloupe mangroves (PMC 106.48, Duperron et al., 2019), water reservoir in Tenerife, Spain (BEA 0247B), inland Tunisia (Osci BM 01, MG762091.1), Salton Sea, California, United States (Dillon et al., 2009), and a shrimp pond in Thailand (TK01; Maa‐iad et al., 2023) where it caused cultured shrimp to die. Although not thermal springs, these habitats are expected to have conditions with high or fluctuating salinity and water level, as well as relatively high temperatures, which corresponds to the conditions in the water of the Xomos spa in Vromoner Canyon (Audy et al., 2022). Although morphologically similar to its most closely related taxa, the novel genus *Xomosiella* does have some differentiating properties. First, the trichomes of *Xomosiella* are highly motile, unlike those of *Persinema* and *Limnothrix*, which are immobile or only slowly trembling. *Lagosinema*, however, is motile (Akagha et al., 2019); therefore, this property might have been merely neglected during the description of *Persinema* (Heidari et al., 2018). In a liquid medium, the filaments moved so intensively that even after shaking into a homogeneous suspension, the biomass entangled itself within a few minutes and formed a compact ball. Unlike other Persinemataceae, some apical cells of *X. audyi* are capitate and slightly bent; fine sheaths are also occasionally present (Figure 1g). These properties, however, were observed only in the cultured strain and not in the natural specimen. Similarly to *Persinema*, *X. audyi* was isolated from a thermal spring, which further differentiated it from brackish *Lagosinema* (Akagha et al., 2019). *Xomosiella audyi* is benthic, crawling over the surface, unlike the true *Limnothrix* distributed in the plankton of colder lakes of the northern hemisphere (Komárek & Anagnostidis, 2008; Meffert, 1971, 1987, 1988; Meffert et al., 1981). Similarly to *X. audyi*, one species of *Limnothrix*, *Lx. lauterbornii* inhabits sulfur‐rich mats, and its trichomes are straight and slowly motile; however, its cells are 2–2.9 (3.8) μm wide whereas in *X*. *audyi*, cells are only up to 2.5 μm wide, and the aerotopes of *Lx. lauterbornii* are positioned centrally (Komárek & Anagnostidis, 2008).

The phylogenetic analysis showed an inflation of the misidentified sequences designated as “*Limnothrix*” (but also other filamentous genera, clade L) clustering with Persinemataceae. The genus *Limnothrix* was originally separated from the genus *Oscillatoria* based on morphology and having one septum per cell, parietal thylakoids, breaks within cells, and mainly polar aerotopes (Meffert, 1987, 1988). A lack of morphological markers caused substantial complications for classical determination of similar morphotypes. The original isolate of *Lx*. *redekei*, the type species of *Limnothrix*, was lost and its sequence is unavailable (Boone & Castenholz, 2001 as cited in Perkerson III et al., 2010), although the short sequence of the strain PCC 9416 (=SAG 3.89) isolated by Meffert from the type locality is identical to the strain NIVA–CYA227/1, today considered its representative type (Guiry & Guiry, 2025; Suda et al., 2002). All these sequences place *Limnothrix* sensu stricto among Pseudanabaenales. Therefore, as already pointed out (Akagha et al., 2019; Duperron et al., 2019), the L clade (Gkelis et al., 2005; Perkerson III et al., 2010; Zhu et al., 2012) will also require the establishment of a new genus at some point in the future. The typification of this clade as the true *Limnothrix* has been suggested (Zhu et al., 2012) but would probably not be justified. Synonymization of *Limnothrix* and *Pseudanabaena* in Pseudanabaenaceae has also been attempted (Whitton, 2011); however, that attempt lacked formal requirements and was, thus, illegitimate (Guiry & Guiry, 2025). The main difference between *Limnothrix* and *Pseudanabaena* was the formation of both polar and central aerotopes only in *Limnothrix*, which was recently disputed after the naming of new species *Ps*. *pruinosa* with centrally and irregularly positioned aerotopes (Aleksovski et al., 2024).

The presence of granules at cross‐walls, easily mistaken for aerotopes, resulted in the designation of the sequences neighboring the genus *Xomosiella* (clade L) as *Limnothrix* spp. (Gkelis et al., 2005; Zhu et al., 2012). In *Limnothrix* sensu stricto, the typical polar granules have been observed as the true aerotopes (Meffert, 1988; Meffert et al., 1981). In the Chinese “*Limnothrix*” strains examined by Zhu et al. (2012) of clade L, the aerotopes were observed in fresh samples, but lost in multiple cultures and not visible under transmission electron microscopy (TEM). Genetically identical strains from Greece were proven, experimentally, to contain true aerotopes; however, their presence was disputed by TEM (Gkelis et al., 2005). Similar granules are characteristic also of Persinemataceae but with equivocal identity. In *Lagosinema*, the granules were visible in light microscopy (LM); however, no TEM observations were completed, and the bodies were hypothesized to contain cyanophycin (Akagha et al., 2019). *Persinema* was diagnosed also on the basis of absent aerotopes; however, some granules were visible within its filaments (Heidari et al., 2018). According to our observations of *Xomosiella* (Figure 1i–k), these bodies were probably composed of cyanophycin that enveloped a carboxysome (Stanier (Cohen‐Bazire), 1988; Walsby, 1994). The typical aerotope structure was not observed, but occasional web‐like structures were present; they were probably fixation artifacts. The additional granules scattered throughout some of the cells in older filaments were possibly polyphosphate granules (Stanier (Cohen‐Bazire), 1988).

### Loriellopsis

Unlike the other cyanobacteria described in this study, the classification of *Loriellopsis vromonerensis* varies between traits. Morphologically, our isolates from crude culture closely resembled *Cyanosarcina*‐like taxa, with the arrangement of polyhedral cells into irregular colonies with thin common mucilage and with the smallest cells of any so far described *Cyanosarcina* species (Komárek & Anagnostidis, 1999; Kováčik, 1988). The sequences from the individually isolated aggregates and the strain clustered congruently; therefore, they represented the same species with possible phenotypic plasticity. Our results also showed that the morphology of the species differed in the unialgal strain in that the colonies disintegrated, and the position of the cells was much looser. This type of variability has been described in numerous coccal taxa (Jezberová & Komárková, 2007; Palinska & Krumbein, 1998). Baeocytes were not observed. Thus, although being coccal without any hint of filaments or heterocytes, the sequences of the *Cyanosarcina*‐like organism Alb16‐JP1 clustered in the immediate neighborhood of *Loriellopsis*, yet only with medium support. They therefore represented a new species of the genus, herein described as *L. vromonerensis*. All the sequences in the *Loriellopsis* clade come from soil, belong to endolithic, epilithic or even directly cave organisms, but are geographically distant from each other: They are from Europe, China, Hawaii, and North America (EF032780.1; JQ404421.1; Lamprinou et al., 2011; Němečková et al., 2023; Shalygin et al., 2019; Smith et al., 2014). This corresponds to the ecology of our isolates from a cave wall, which is probably a typical habitat for the entire group. Inhabiting endolithic and soil cavities as well as caves in combination with protective pigments is possibly also a strategy to protect the cells from UV radiation (Němečková et al., 2023, 2024).

*Loriellopsis cavernicola*, the type and, heretofore, only species of the genus, was originally placed in the today‐obsolete family Symphyonemataceae in Nostocales (Lamprinou et al., 2011), which was recently merged with Scytonemataceae (Strunecký et al., 2023). In our analyses, the genus had unclear but apparently distant relationships with the rest of both “Symphyonemataceae” (represented here by *Iphianassa*) and Stigonemataceae, in agreement with previous authors (Kilgore et al., 2018; see Figure S2). Conversely, Chroococcidiopsidales became infamous for constantly coming out polyphyletic or paraphyletic in multiple sources (Jung et al., 2020, 2021; Wang et al., 2019). So, how is it possible that a heterocystous genus is so closely related to coccals?

- Due to phenotypic plasticity, we captured only one life stage of our *Loriellopsis vromonerensis* isolate, which might, under some conditions, transit into a filamentous, heterocytous form. *Loriellopsis cavernicola* has formed short hormocysts (Lamprinou et al., 2011); therefore, a larger “coccal” stage is possible. In Nostocales, polymorphic cycles including nearly coccal stages have been known in *Cyanocohniella* (Jung et al., 2020; Kaštovský et al., 2014). *Umezakia natans*, a true‐branching species, was revealed to be merely an environmentally induced morphotype of an isopolar *Chrysoporum ovalisporum*, in which the true branching was induced by vitamin deficiency (McGregor et al., 2023). This explanation is probably incorrect: We have observed the organism under different culture conditions and for long periods of time, and not once has a stage other than the bundle‐like structure been documented. Furthermore, the thylakoids of Alb16‐JP1 were parietal, unlike the parallel or fascicular forms of *L*. *cavernicola* (Lamprinou et al., 2011).
- The original sequence of the *Loriellopsis* reference may have been contaminated. Although the reference sequence is designated as the type strain of *L*. *cavernicola*, the original publication did not mention sequencing of the strain itself; instead, the material for DNA extraction was gathered by scraping from the cave wall and was neither cultured nor further isolated (Lamprinou et al., 2011). Therefore, there is no guarantee of matching the sequence to the phenotype. Interestingly, there are other sequences of uncultured “*Loriellopsis*” deposited in NCBI, with sequence similarity to multiple completely isolated lineages including *Kovacikia* (Kilgore et al., 2018) or even *Micractinium* and higher plant plastids. We have not been able to obtain the original material of *Loriellopsis*, so this possibility remains unproven, but we consider it highly probable. Contamination of apparently unicyanobacterial natural material by smaller inconspicuous species is a very common phenomenon. It is also true that DNA can sometimes be isolated much more easily from these smaller species with thinner mucilaginous sheaths.
- The convergent evolution and homoplastic mutations of the 16S rRNA gene in *Loriellopsis cavernicola* have brought about misleading results of phylogenetic analyses (Dvořák et al., 2023). In this case, using only 16S rRNA gene data may be insufficient (Skoupý et al., 2024). However, this problem is much broader than the content of this article. Further sampling efforts, preferably genomic data from morphologically authentic unialgal strains, might resolve this issue in the future.

The general morphotype mostly lacking reliable morphological markers makes the identification of *Cyanosarcina*‐ or *Chroococcidiopsis*‐like cyanobacteria even more complicated. This situation has also resulted in inflation of sequences belonging to both mentioned genera with clearly polyphyletic relationships (Figure 9). Because of the unrelatedness to other genera, we believe that the cleanest solution to this situation is to describe these organisms as a new genus from the order Chroococcidiopsidaceae.

### Mastigocladus

The results of the phylogenetic analysis showed that the strain morphologically corresponding to the genus *Mastigocladus* (Hapalosiphonaceae), with the true branching and long attenuated branches and intercalary heterocytes (Komárek, 2013), truly represented a separate species belonging to this genus. Its position as sister to all other *Mastigocladus* sequences was well supported statistically, and the genetic distance was sufficient to describe it as a new species, although not high enough to be an isolated genus (Casamatta et al., 2020; Yarza et al., 2014).

For a long time, *Mastigocladus laminosus* was the only validly described species of the genus, albeit with a number of supraspecific taxa arguably belonging to different genera (Komárek, 2013). Recently, Jaiswal et al. (2021) described a genetically and morphologically distinct species (*M*. *ambikapurensis*) from hot spring water (52°C) differing also by the presence of both terminal and lateral heterocytes. The herein described *M*. *boudae* possessed only intercalary heterocytes; therefore, this phenotypic marker might be useful for further identification. Admittedly, the very presence and position of heterocytes may be induced by the culture conditions (Nierzwicki‐Bauer et al., 1984; Tiwari, 1978). There are few other morphologically described yet unresolved species of the genus. *Mastigocladus bursa* was described very vaguely with little morphological information (elliptic cells, attenuated branches, bright green coloring, young filaments resemble *Nostoc*) from a mountain stream in Mauritius (Dickie, 1874; Forti, 1907). *Mastigocladu*s *flagelliforme* (*Chondrogloea flagelliformis*) was somewhat similar to *M*. *boudae*, with the distinctly constricted cells and the dimensions of the main filament; however, the branches of *M*. *boudae* were at least 5 μm wide, whereas those of *Ch*. *flagelliformis* were only 2 μm wide. Furthermore, *Ch*. *flagelliformis* had only one‐sided branching, whereas *M*. *boudae* branched in multiple directions (Komárek, 2013; Schmidle, 1900). *Mastigocladus hansgirgii* did not possess true branching and is probably a member of a different genus (Komárek, 2013; Schmidle, 1900). *Mastigocladus major* (*Hapalosiphon major*) also had quite unclear morphological descriptions and has been considered just a morphological form of *M. laminosus* (Elenkin, 1936). Soe et al. (2011) reported multiple lineages within *M*. *laminosus* in agreement with our analyses and considered them all as members of the same species with morphological differences congruent with geographical isolation. With more data, it is possible that some of these lineages will be erected as new species or perhaps connected to the other morphologically described taxa.

Frequent morphological similarities among genera in certain life stages in all Hapalosiphonaceae combined with phenotypic plasticity have led to the inflation of misidentified sequences (Parmar et al., 2024), which was also apparent in our analyses. Furthermore, the usage of 16S rRNA gene similarity thresholds has been problematic within this family, as many genera have quite low 16S rRNA gene differences (Table 3), and thus, the 16S rRNA gene p‐distance should not be the sole evidence for species and genus delineation (Casamatta et al., 2020). For example, *Neowestiellopsis*, *Reptodigitus*, *Nostochopsis*, and *Pelatocladus* should, theoretically, belong to a single genus; however, the putative taxon would be paraphyletic, which would be in conflict with a monophyletic species concept (Johansen & Casamatta, 2005). Therefore, all available characteristics, including ecology and molecular markers, ought to be used for more reliable identification (Casamatta et al., 2020).

The taxonomical situation between *Mastigocladus* and *Fischerella* has not been satisfactorily resolved to date (Alcorta et al., 2019). The genera share identical locus classicus and possibly even the original population set as a type (Bornet & Flahault, 1886; Elenkin, 1938; Gomont, 1895; Kaštovský et al., 2014; Kaštovský & Johansen, 2008; Kirchner, 1898; Schwabe, 1837). Both type species reportedly differed in cell size, which was considered irrelevant by Rippka et al. (1979) who treated the genera as synonymous under the name *Fischerella*, taking its taxonomic priority into account (Gomont, 1895; Kirchner, 1898). Later authors synonymized the genera even further with *Hapalosiphon* and *Westiellopsis* (Gugger & Hoffmann, 2004). Kaštovský and Johansen (2008) following Geitler (1932) argued that specimens from thermal habitats should be considered the true *Mastigocladus*, whereas other morphologically similar species from different habitats should be considered *Fischerella*. According to Komárek (2013), the main filaments of *Fischerella* may be polyseriate, further distinguishing the two taxa. The clade including our strain complied with these rules, as all isolates of the genus for which there are available data have come from thermal environments and lack polyseriate filaments (Alcamán et al., 2017; Dagan et al., 2013; Hirose et al., 2016; Jaiswal et al., 2021; Kaštovský & Johansen, 2008; Miller et al., 2007; Mishra et al., 2020; Roeselers et al., 2007; Smith‐Bädorf et al., 2013; Soe et al., 2011; Yilmaz Cankilic & Arik Berk, 2016; Zhu et al., 2017). Therefore, the entire clade ought to be regarded as *Mastigocladus* in agreement with previous authors (Jaiswal et al., 2021; Kaštovský & Johansen, 2008).

### Pegethrix

The phylogenetic analysis placed the novel strain Alb13‐JP2 within the genus *Pegethrix* (Oculatellales), while the genetic distance supported its description as a separate species (lowest 16S rRNA gene PD = 2.51%, to *Pegethrix niliensis*). This correlated with its morphology, as it produced simple uniseriate filaments without conspicuous morphological properties (Gao et al., 2024; Hentschke et al., 2024; Luz et al., 2023; Mai et al., 2018). The species is so far represented by sequences of three strains. The identity of one of these strains (“*Leptolyngbya*” sp. PltLPT2) was already discussed by Tang et al. (2021), who considered it part of the invalidly described genus *Marsacia* (Brown et al., 2010). The strain PltLPT2 originated from a Greek thermal spring (Bravakos et al., 2016) and, therefore, shares ecological characteristics and geographical region with our isolate (Alb13‐JP2) of *P*. *sulphurea*. The third strain within *P*. *sulphurea*, “*Leptolyngbya*” sp. WUC607, was collected in Punjab wetlands (ON685867.1). Compared to most other species of *Pegethrix*, *P*. *sulphurea* morphologically differs by the absence of false branching, an absence which has, so far, been reported only in *P*. *niliensis* (Gao et al., 2024; Hentschke et al., 2024; Luz et al., 2023; Mai et al., 2018; Shen et al., 2023). Almost all species of *Pegethrix*, apart from planktonic *P*. *qiandaoensis*, grow on moist terrestrial surfaces (Gao et al., 2024; Hentschke et al., 2024), whereas *P*. *sulphurea* inhabits a highly sulfuric thermal environment. However, affinity for thermal habitats is not rare among Oculatellaceae and appears to have evolved without a clear evolutionary pattern. Typically, thermophilic are members of the genus *Thermoleptolyngbya* (Sciuto & Moro, 2016; Tang et al., 2021), the monospecific genera *Komarkovaesiopsis* (Cai et al., 2024) and *Amphirytos* (Jasser et al., 2022), and the species *Albertania pratii* and *Oculatella castenholzii* (Kaštovský et al., 2023).

It is plausible that our isolate belongs to an already described thermophilic or thermotolerant species that lacks molecular data (Mai et al., 2018); however, morphologically, none fully matched our strain. Usually, the main difference was in the cell length‐to‐width ratio or in that, the cells were not constricted at cross‐walls. The cells of, for example, *Leptolyngbya copelandii or Lyngbya mucicola* var. *hiraoensis* are similarly wide but at least twice as long, and those of *Leptolyngbya delicatula* are similarly long, constricted at cross‐walls, but half as wide (Anagnostidis, 2001; Compère, 1985; Komárek & Anagnostidis, 2008). Reports of *Drouetiella lurida* (as *Phormidium luridum*) from thermal habitats (Copeland, 1936) may belong to another similar species, as its type material was from fresh water and soil (Gomont, 1892; Mai et al., 2018). *Leptolyngbya valderiana*, which has longer cells and confluent sheaths, was also observed in colder freshwater habitats (Copeland, 1936).

*Heteroleibleinia kuetzingii*, *H. affixa*, and *Leibleinia epiphytica* were considered thermotolerant, albeit adherent to or creeping on the substrate (Copeland, 1936; Komárek & Anagnostidis, 2008; Yoneda, 1938); however, this trait might be influenced by culture conditions. Our strain lacked the typical fascicles of *Symploca nemecii*, trichomes of which are constricted at cross‐walls, and with slightly thinner cells than what we observed in *P*. *sulphurea* (Copeland, 1936; Prát, 1929). *Leptolyngbya thermophila* has longer cells and possesses attenuated apex cells and was described from hot springs in Kamchatka (Elenkin, 1914; Komárek & Anagnostidis, 2008). The most morphologically and eco‐geographically similar to *Pegethrix sulphurea* is arguably *Leptolyngbya thermobia*, described from Greece, with trichomes constricted at cross‐walls and nearly identical cell dimensions (Anagnostidis, 2001). Although our isolate did not form spirals typical for *Leptolyngbya thermobia*, this phenotypic trait might also be altered by culture conditions. Molecular evidence from the authentic material of these species is essential in order to elucidate their possible phylogenetic relationships.

The emergence of numerous new taxa of a simple trichal morphotype has been a pattern of the past several years (Kaštovský, 2023) and has ultimately led to the establishment of four orders, Leptolyngbyales, Nodosilineales, Oculatellales, and Pseudanabaenales, predominantly represented by this morphotype (Strunecký et al., 2023) and originally included in Synechococcales sensu Komárek et al. (2014). Since the relatively recent description of *Oculatella* (Zammit et al., 2012) and the subsequent erection of the family Oculatellaceae (Mai et al., 2018) and the order Oculatellales (Strunecký et al., 2023), respectively, nearly 70 new species in 23 genera have been described from a variety of habitats (Guiry & Guiry, 2025). However, many originated in the earlier established collective genera, such as *Leptolyngbya* (Mai et al., 2018). In fact, one third of newly described or renamed species since the beginning of this century have belonged to simple trichal morphotypes (Kaštovský, 2023). Oculatellaceae, especially the clade containing closely related *Carthusia*, *Drouetiella, Elainella, Pegethrix*, *Maricoleus*, and *Siamcapillus*, are infamous for significant rates of cryptic and pseudocryptic diversity (Hentschke et al., 2024), and molecular evidence has been the most reliable for distinguishing Oculatellaceae taxa from each other (Osorio‐Santos et al., 2014). Our results, therefore, align with previous authors, who have suggested merging the aforementioned genera into one (Hentschke et al., 2024) because in this clade, no two strains, excluding *Drouetiella*, cross the 16S rRNA gene PD threshold for separate genera (Tables S4 and S5; Yarza et al., 2014).

## AUTHOR CONTRIBUTIONS

**Jan Pokorný:** Investigation (equal); resources (supporting); writing – review and editing (equal). **Alžběta Vondrášková:** Investigation (equal); writing – review and editing (equal). **Michaela Wipplingerová:** Investigation (supporting); resources (supporting). **Jan Kaštovský:** Conceptualization (lead); formal analysis (equal); funding acquisition (lead); investigation (equal); project administration (lead); supervision (lead); writing – original draft (equal); writing – review and editing (equal).

## Supporting information


**Figure S1.** The phylogenetic tree constructed from the 16S rRNA gene sequences of 203 taxa, 1096 positions long, showing the phylogenetic position of *Xomosiella* in Persinemataceae (Nodosilineales) with five sequences of Gloeobacterales applied as an outgroup. Representatives of Chroococcales, Leptolyngbyales, Acaryochloridales, Thermostichales, Synechococcales, Gloeomargaritales, and Pseudanabaenales were also included in the analyses. The topology represents the best ML tree with the best model GTR + R chosen by SMS. Node support includes the bootstrap of ML analysis above 50 and posterior probabilities of the BI analysis above 0.95. Our four sequences of *X*. *audyi* are highlighted in bold.

**Figure S2.** The phylogenetic tree constructed from the 16S rRNA gene sequences of 189 taxa, 1096 positions long, showing the phylogenetic position of *Loriellopsis vromonerensis* with five sequences of Gloeobacterales applied as an outgroup. Representatives of Chroococcales, Pleurocapsales, and Nostocales were also included in the analyses. The topology represents the best ML tree with the best model GTR + R chosen by SMS. Node support includes the bootstrap of ML analysis above 50 and posterior probabilities of the BI analysis above 0.95. Our six sequences of *L. vromonerensis* are highlighted in bold.

**Figure S3.** The phylogenetic tree constructed from the 16S rRNA gene sequences of 106 taxa, 1096 positions long, showing the phylogenetic position of *Mastigocladus boudae* in Hapalosiphonaceae with eight sequences of Chroococcidiopsidales applied as an outgroup. Other representatives of Nostocales were also included in the analyses. The topology represents the best ML tree with the best model GTR + R chosen by SMS. Node support includes the bootstrap of ML analysis above 50 and posterior probabilities of the BI analysis above 0.95. Our four sequences of *M*. *boudae* are highlighted in bold.

**Figure S4.** The phylogenetic tree constructed from the 16S rRNA gene sequences of 145 taxa, 1157 positions long, showing the phylogenetic position of *Pegethrix sulphurea* within Oculatellaceae with one sequence of Gloeobacterales applied as an outgroup. Representatives of Nodosilineales and Leptolyngbyales were also included in the analyses. The topology represents the best ML tree with the best model GTR + R chosen by SMS. Node support includes the bootstrap of ML analysis above 50 and posterior probabilities of the BI analysis above 0.95. Our four sequences of *P*. *sulphurea* are highlighted in bold.

**Table S1.** Percent 16S rRNA gene sequence dissimilarity (p‐distance) of *Xomosiella audyi* and related taxa.

**Table S2.** Percent 16S rRNA gene sequence dissimilarity (p‐distance) of *Loriellopsis vromonerensis* and related taxa.

**Table S3.** Percent 16S rRNA gene sequence dissimilarity (p‐distance) of *Mastigocladus boudae* and related taxa in Hapalosiphonaceae.

**Table S4.** Range in percent 16S rRNA gene sequence dissimilarity (p‐distance) of *Pegethrix suplhurea* and related taxa in Oculatellaceae, based on a distance of all OTUs within a clade to all OTUs in the comparison clade.

**Table S5.** Percent 16S rRNA gene sequence dissimilarity (p‐distance) of *Pegethrix sulphurea* and related taxa in Oculatellaceae.
