# Supplementary figures and images for: Four novel taxa of cyanobacteria from a unique thermal cave habitat in Vromoner Canyon, Albania
Source: J Phycol. 2025 Sep 12;61(5):1394–422. doi: 10.1111/jpy.70082 (PMC12547637; doi:10.1111/jpy.70082)

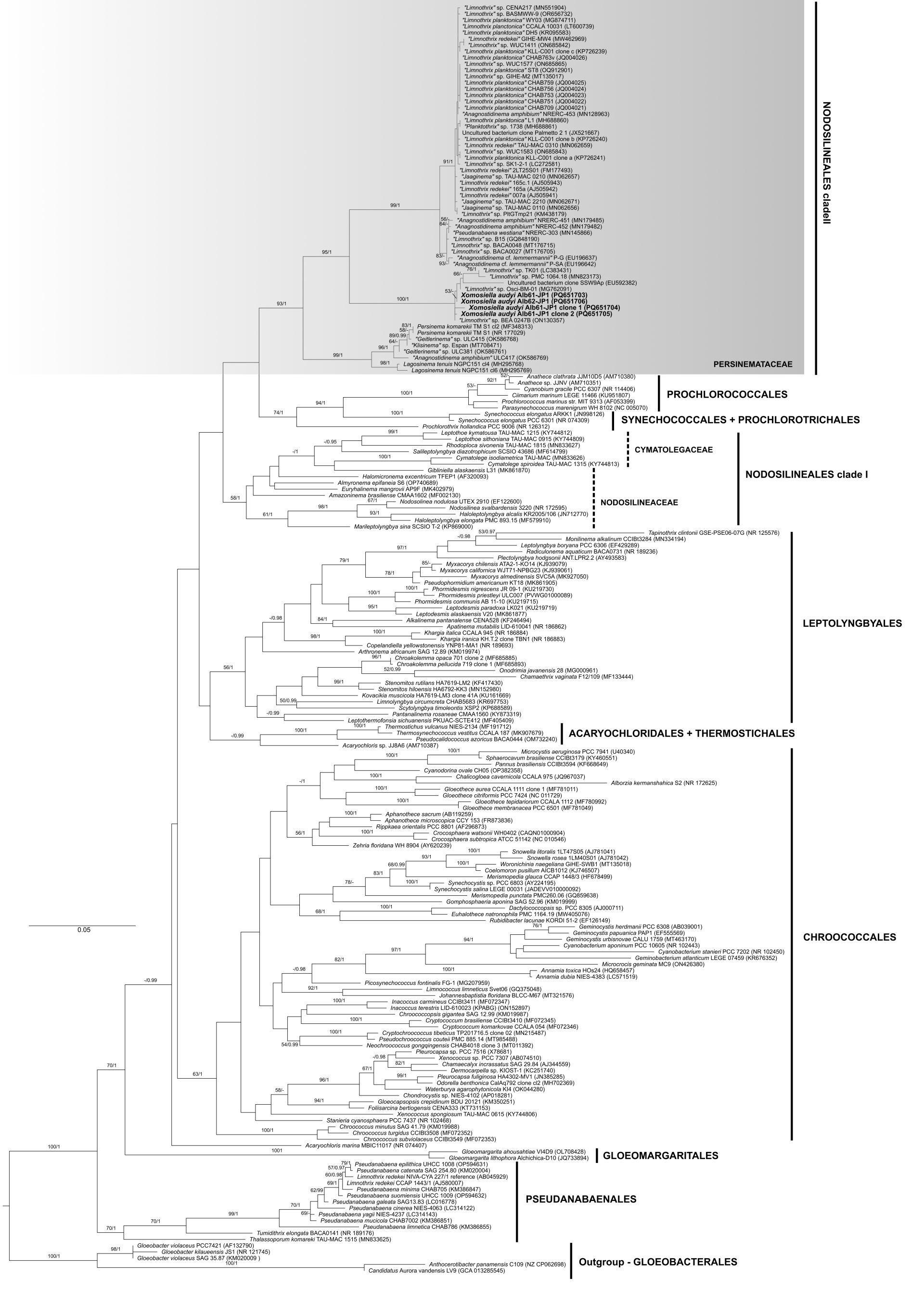

Supplement: Supplementary file 1 — Figure S1. The phylogenetic tree constructed from the 16S rRNA gene sequences of 203 taxa, 1096 positions long, showing the phylogenetic position of Xomosiella in Persinemataceae (Nodosilineales) with five sequences of Gloeobacterales applied as an outgroup. Representatives of Chroococcales, Leptolyngbyales, Acaryochloridales, Thermostichales, Synechococcales, Gloeomargaritales, and Pseudanabaenales were also included in the analyses. The topology represents the best ML tree with the best model GTR + R chosen by SMS. Node support includes the bootstrap of ML analysis above 50 and posterior probabilities of the BI analysis above 0.95. Our four sequences of X. audyi are highlighted in bold. [file JPY-61-1394-s007.tif]

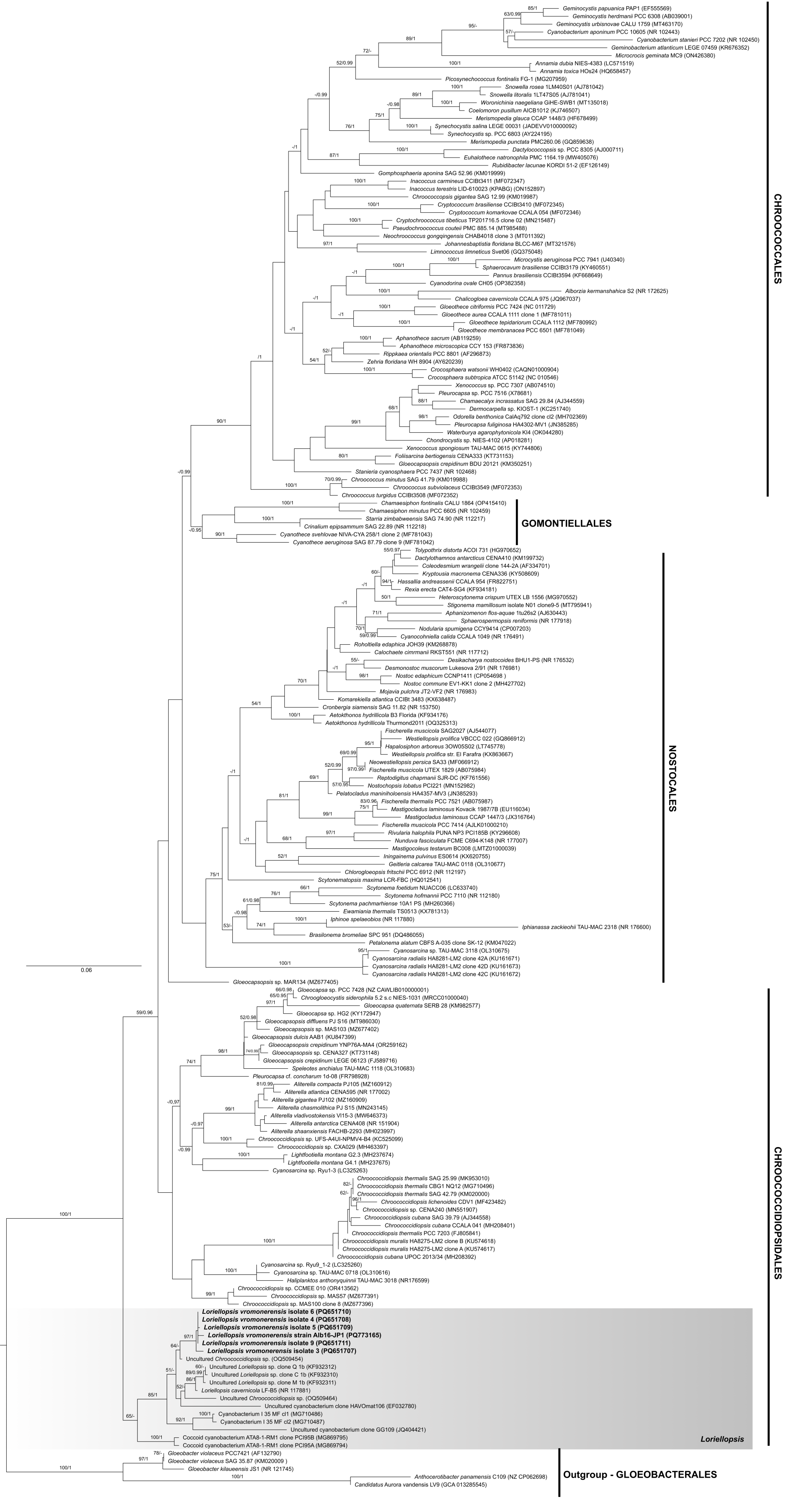

Supplement: Supplementary file 2 — Figure S2. The phylogenetic tree constructed from the 16S rRNA gene sequences of 189 taxa, 1096 positions long, showing the phylogenetic position of Loriellopsis vromonerensis with five sequences of Gloeobacterales applied as an outgroup. Representatives of Chroococcales, Pleurocapsales, and Nostocales were also included in the analyses. The topology represents the best ML tree with the best model GTR + R chosen by SMS. Node support includes the bootstrap of ML analysis above 50 and posterior probabilities of the BI analysis above 0.95. Our six sequences of L. vromonerensis are highlighted in bold. [file JPY-61-1394-s005.tif]

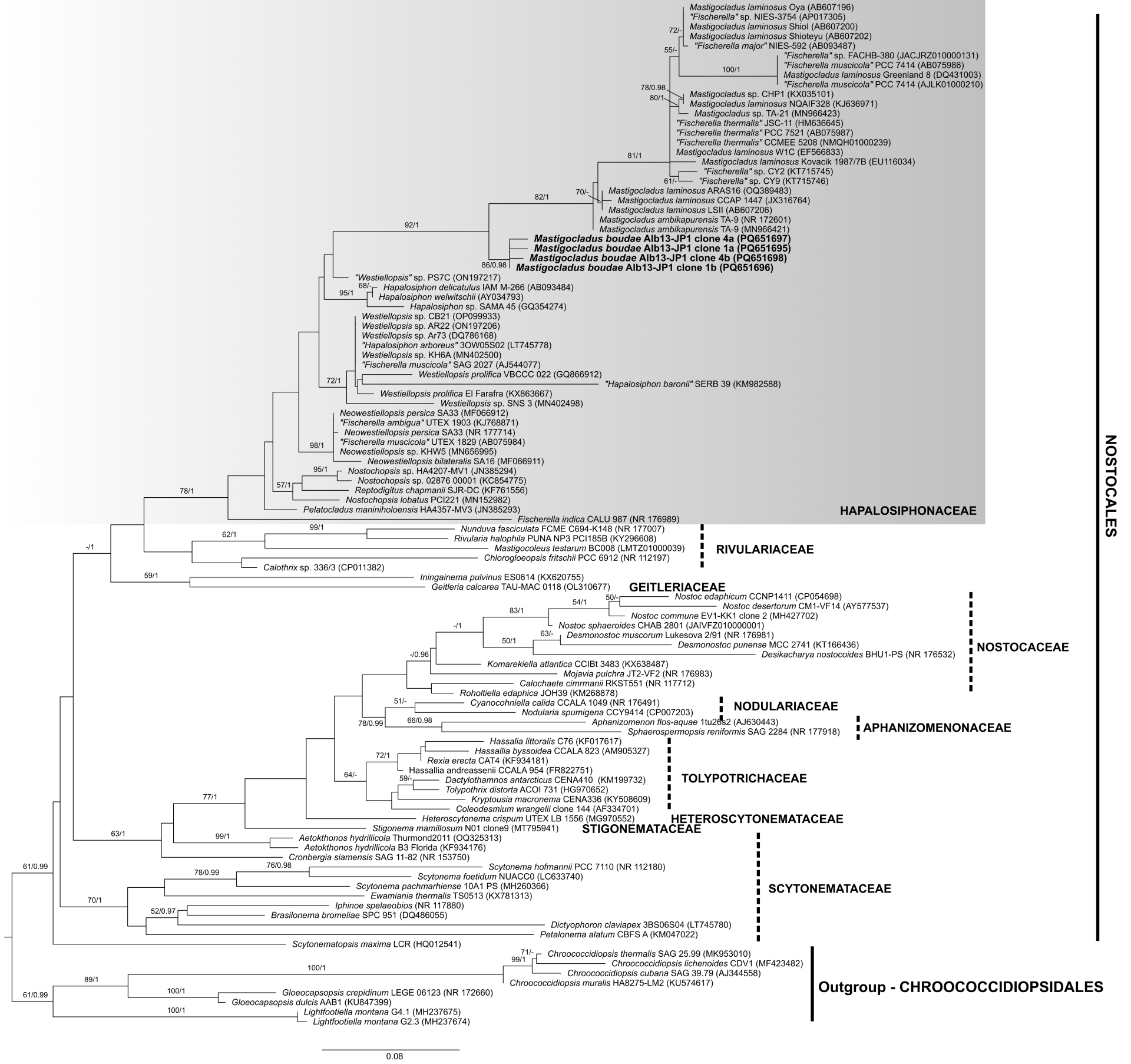

Supplement: Supplementary file 3 — Figure S3. The phylogenetic tree constructed from the 16S rRNA gene sequences of 106 taxa, 1096 positions long, showing the phylogenetic position of Mastigocladus boudae in Hapalosiphonaceae with eight sequences of Chroococcidiopsidales applied as an outgroup. Other representatives of Nostocales were also included in the analyses. The topology represents the best ML tree with the best model GTR + R chosen by SMS. Node support includes the bootstrap of ML analysis above 50 and posterior probabilities of the BI analysis above 0.95. Our four sequences of M. boudae are highlighted in bold. [file JPY-61-1394-s009.tif]

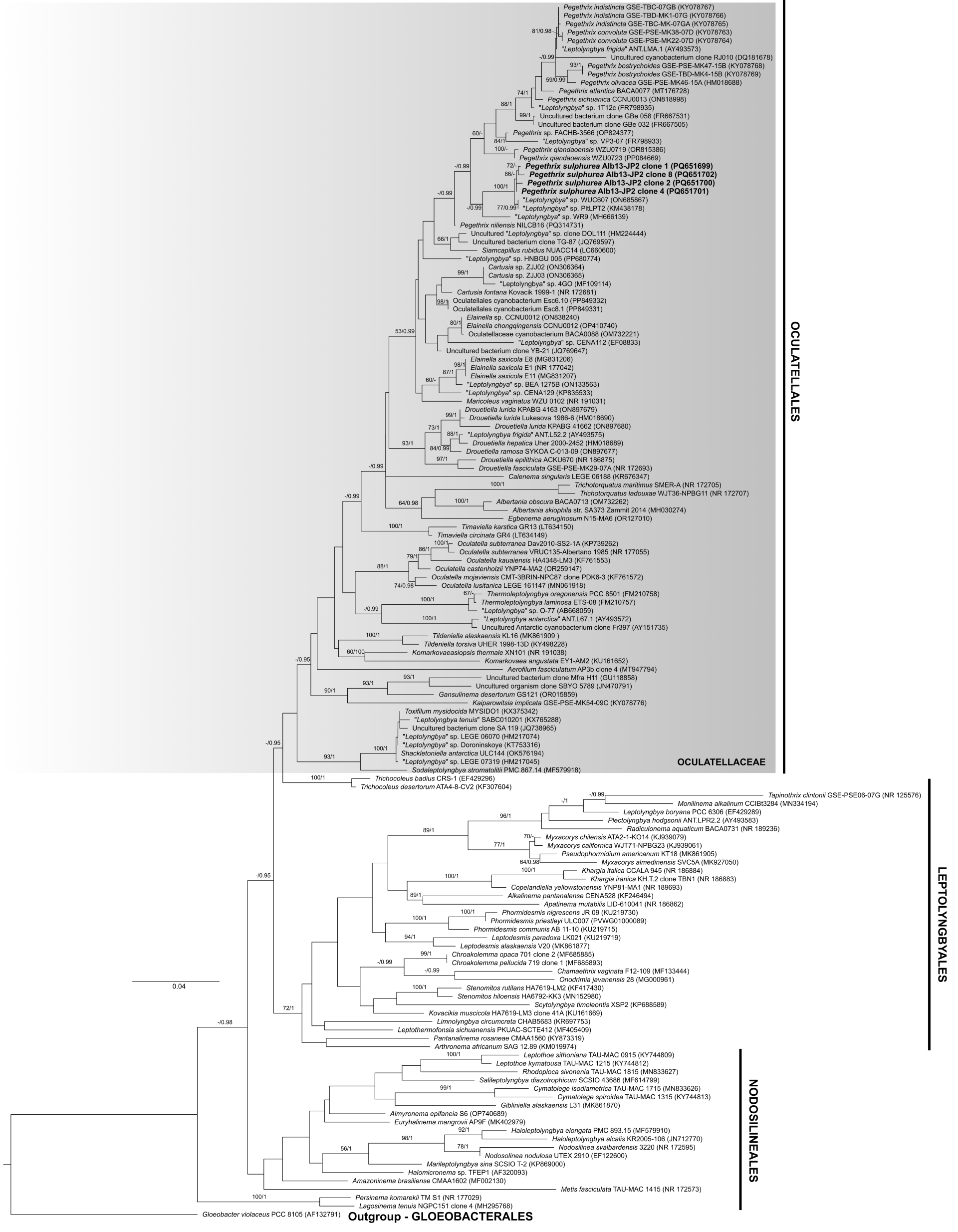

Supplement: Supplementary file 4 — Figure S4. The phylogenetic tree constructed from the 16S rRNA gene sequences of 145 taxa, 1157 positions long, showing the phylogenetic position of Pegethrix sulphurea within Oculatellaceae with one sequence of Gloeobacterales applied as an outgroup. Representatives of Nodosilineales and Leptolyngbyales were also included in the analyses. The topology represents the best ML tree with the best model GTR + R chosen by SMS. Node support includes the bootstrap of ML analysis above 50 and posterior probabilities of the BI analysis above 0.95. Our four sequences of P. sulphurea are highlighted in bold. [file JPY-61-1394-s004.tif]
